# Revealing CO_2_-Fixing SAR11 Bacteria in the Ocean by Raman-Based Single-Cell Metabolic Profiling and Genomics

**DOI:** 10.34133/2022/9782712

**Published:** 2022-10-13

**Authors:** Xiaoyan Jing, Yanhai Gong, Teng Xu, Paul A. Davison, Craig MacGregor-Chatwin, C. Neil Hunter, La Xu, Yu Meng, Yuetong Ji, Bo Ma, Jian Xu, Wei E. Huang

**Affiliations:** ^1^Single-Cell Center, CAS Key Laboratory of Biofuels, Shandong Key Laboratory of Energy Genetics and Shandong Institute of Energy Research, Qingdao Institute of Bioenergy and Bioprocess Technology, Chinese Academy of Sciences, Qingdao, Shandong, China; ^2^Laboratory for Marine Biology and Biotechnology, Qingdao National Laboratory for Marine Science and Technology, Qingdao, Shandong, China; ^3^University of Chinese Academy of Sciences, Beijing, China; ^4^Plants, Photosynthesis and Soil, School of Biosciences, University of Sheffield, Sheffield S10 2TN, UK; ^5^Disease and Fishery Drugs Research Center, Marine Biology Institute of Shandong Province, Qingdao, ShandongChina; ^6^Single-Cell Biotechnology, Ltd, Qingdao, ShandongChina; ^7^Department of Engineering Science, University of Oxford, Parks Road, OX1 3PJ Oxford, UK

## Abstract

The majority of marine microbes remain uncultured, which hinders the identification and mining of CO_2_-fixing genes, pathways, and chassis from the oceans. Here, we investigated CO_2_-fixing microbes in seawater from the euphotic zone of the Yellow Sea of China by detecting and tracking their ^13^C-bicarbonate (^13^C-HCO_3_^-^) intake via single-cell Raman spectra (SCRS) analysis. The target cells were then isolated by Raman-activated Gravity-driven Encapsulation (RAGE), and their genomes were amplified and sequenced at one-cell resolution. The single-cell metabolism, phenotype and genome are consistent. We identified a not-yet-cultured *Pelagibacter* spp., which actively assimilates ^13^C-HCO_3_^-^, and also possesses most of the genes encoding enzymes of the Calvin-Benson cycle for CO_2_ fixation, a complete gene set for a rhodopsin-based light-harvesting system, and the full genes necessary for carotenoid synthesis. The four proteorhodopsin (PR) genes identified in the *Pelagibacter* spp. were confirmed by heterologous expression in *E. coli*. These results suggest that hitherto uncultured *Pelagibacter* spp. uses light-powered metabolism to contribute to global carbon cycling.

## 1. Introduction

Photosynthesis is a fundamental biological process, which uses solar energy to power the fixation of CO_2_. This process of photoautotrophy is responsible for nearly all of the Earth’s primary production of 104.9 petagrams of carbon per year (Pg of C/yr), nearly half of which (48.5 Pg of C/yr) is accounted for by photosynthesis in the oceans [1]. Since most marine bacteria are not yet cultured [2, 3], they play an enigmatic yet important role in terms of photosynthesis and CO_2_ fixation.

Two distinct systems have been discovered so far for harvesting solar energy: chlorophyll-based photochemistry and rhodopsin-based, light-activated proton pumps [4, 5]. Chlorophyll-based systems in cyanobacteria have been studied intensively for decades, and the importance of rhodopsin-based systems has become increasingly clear; for example, a recent survey suggested that microbial rhodopsins are major contributors to solar energy harvesting in the oceans [6]. Although chlorophyll-based photosynthesis involves the assembly of a large, relatively complex network of light-harvesting and charge-separating chlorophyll-protein complexes, there is a greater energetic benefit obtained than that from a rhodopsin-based mechanism [7]. However, the simple and minimal cost of synthesising proteorhodopsin has made it globally abundant in microbes [5, 8–12]. It has also been found that proteorhodopsin (PR) can simulate bacterial growth [13] or support bacteria surviving in poor nutrient conditions [14, 15]. Thus, due to its ubiquitous distribution and high abundance, rhodopsin-based phototrophy plays an important role in nature [16, 17]. Chlorophyll- and rhodopsin-based systems absorb different and complementary wavelengths of the solar spectrum [6] and were thought to rarely coexist in a host. However, recent studies have shown that these two distinct systems can exist in one single bacterium (e.g., *Tardiphaga* sp.) for enhanced solar energy utilization [18, 19].

Chlorophyll-based photosynthesis is a well-known light-harvesting system for CO_2_ fixation [1]. PR-based photosynthesis such as *Dokdonia* sp. MED134 has been reported to fix CO_2_ in the presence of light [13]. Subsequently, anaplerotic CO_2_ fixation in marine bacteria has been reported [20–28]. PR-containing bacteria could be the most abundant [29, 30], with microbial rhodopsins making a large contribution to solar energy harvesting in the oceans [6]. However, it is still unknown whether PR-containing bacteria in natural conditions can fix CO_2_.

Single-cell Raman microspectroscopy, a label-free technique to measure molecular vibrational profiles of single cells, offers a powerful suite of approaches for correlating cellular function and genomics [10, 31–37]. Single-cell Raman spectra (SCRS) display the intrinsic biochemical fingerprints of individual cells, which can be used as a form of metabolic phenotype [38, 39]. When coupled with stable isotope probing (Raman-SIP), SCRS can be used to measure specific functions of cell metabolism, exploiting the shifts in some Raman bands when bacteria incorporate stable isotopes (e.g., ^13^C, ^15^N, and ^2^H) ([31, 39–43]; Hatzenpichler et al.; [34]). Subsequently, individual cells displaying particular bacterial metabolic changes can be selected for genomic analysis using Raman-activated cell sorting (RACS) techniques [32, 44, 45], such as Raman tweezers [38, 46], Raman-activated microfluidic sorting (RAMS) [47–49], Raman-activated droplet sorting (RADS) [50], and Raman-activated cell ejection (RACE) [10, 36, 51, 52].

One challenge of Raman-based single-cell genomics is that genome coverage is usually low (<20%), providing limited genotypic information about the targeted cells. Although minimetagenomics from a small number of sorted cells (rather than one single cell) and binning technology can improve genome coverage [53, 54], they introduce uncertainty in assigning the correlated genomic and functional information to specific cells [10, 36].

In this study, we applied Raman-activated Gravity-driven Encapsulation (RAGE) [55] to precisely isolate CO_2_-fixing bacteria, one cell at a time. Single bacterial cells from the euphotic zone of the Yellow Sea of China were incubated with ^13^C-HCO_3_^-^, then sorted and whole-genome sequenced based on the Raman shifts in SCRS, which were altered as a result of incorporating ^13^C. We isolated a strain of *Pelagibacter* spp. able to fix ^13^C-HCO_3_^-^. It suggests that PR-containing bacteria should contribute to solar energy harvesting in the oceans and could also play an important role in the CO_2_ global budget.

## 2. Materials and Methods

### 2.1. Seawater Sampling and Preparation

Seawater was sampled at the euphotic zone in an inshore site located in a fishery area of Laoshan Bay in the Yellow Sea, China (Figure S1). The euphotic water samples were collected for the subsequent experiments involving incubation with different carbon sources and identification of carotenoid-containing bacteria. The seawater was filtered through a 3 *μ*m membrane to collect microbial biomass.

Five sets of seawater samples were treated with different carbon sources separately, including original seawater (“C-free” for short), ^12^C-NaHCO_3_, ^13^C-NaHCO_3_, ^12^C-CO_2_, and ^12^C-CO_2_ plus ^13^C-NaHCO_3_ (“C-combined” for short), all in triplicate. Among them, the C-free treatments serve as an incubation control. The final concentration of the total carbon source (^12^C or ^13^C bicarbonate) was 2 mM. For the treatment group with CO_2_, the seawater was bubbled with 0.5% CO_2_. Seawater microbes were kept in glass bottles in the open air and under constant natural light conditions. Firstly, after pretreatment, the cells were filtered by a 0.22 *μ*m pore-size membrane and then frozen at -80°C until DNA extraction for the following high-throughput 16S rRNA sequencing. Secondly, during incubation, cells in samples with different carbon sources were separately enriched by passing a ~70 ml sample through a Centricon® Plus-70 Ultracel PL-100 centrifugal filter (Merck Millipore, USA). After that, the cells were partly harvested for Raman measurement based on the timeline and partly used for Raman-based single-cell sorting according to the utilization function of ^13^C-NaHCO_3_.

As a negative control, dark incubation experiments were carried out for the seawater samples. Specifically, the seawater samples were, respectively, incubated with ^12^C-NaHCO_3_ and ^13^C-NaHCO_3_, each in triplicates, with the bottles wrapped in tin foil. All incubation conditions and SCRS acquisition setting are identical to the experiments above.

### 2.2. Chip for Raman-Based Single-Cell Sorting

To decrease Raman background noise, the RAGE chip is made of quartz. The chip consists of two quartz slides: an upper layer with inlet holes and open wells and a bottom layer consisting of a microchannel for cells. The microchannel on the bottom layer is built via the wet-etching method. Specifically, a 2 *μ*m positive photoresist layer is spined on a quartz slide with 100 nm chromium. Then, the slide is covered with a photomask of the microchannel structure and exposed to UV light. The channel is created after etching with chromium etchant (Ce(NH_4_)_2_(NO_3_)_6_ in HClO_4_ solution) and quartz etchant (HF-HNO_3_ solution). The bottom layer with microchannel is then cleaned with acetone and bonded with the upper layer at 100°C. Finally, the quartz chip is treated with dichlorodimethylsilane for hydrophobic decorating, for generating the microdroplets of the proper size.

Single-cell Raman analysis is conducted in the detection window on the chip. The distance from the detection window to the oil well is about 500 *μ*m. A sorting channel connected the detection window and oil well. The scale of the thin sorting channel is adjusted for different sizes of cells. We usually use a ~30 *μ*m width and 10 *μ*m depth microchannel for the sorting of microbial cells and ~50 *μ*m width and 30 *μ*m depth for the sorting of microbial cells.

### 2.3. Single-Cell Raman Microspectroscopy

All the single-cell Raman spectra were acquired on a RACS-Seq system (Qingdao Single-cell Biotech, China) or a LabRam HR system (Horiba, France). The spectra were analyzed with LabSpec 6 software and customized scripts. The Raman bands in SCRS from carotenoid-containing cells were determined and further analyzed to establish a relationship between the significant Raman shifts and ^13^C absorption. The Raman bands in SCRS of carotenoid-containing cells from a ^12^C-labeled sample were used as controls. Cells with a ^13^C shift in SCRS were isolated using the RAGE chip as described above. After that, the tube which contained the target cells in a one-cell-one-tube manner was then moved into a laminar hood, and lysis buffer (Qiagen, USA) was added for the following cell lysis.

### 2.4. Proteorhodopsin Overexpression and Purification

Four candidate proteorhodopsin genes identified from RG1 and RG6 were chemically synthesised (Integrated DNA Technologies, USA) with a C-terminal 6xHis tag and flanked by *Nco*I and *Pme*I restriction enzyme sites at the N- and C-terminus, respectively. These 4 genes were ligated into the arabinose-inducible pBADMycHisA vector, their integrity confirmed by DNA sequencing (Eurofins Genomics, Germany) and the resulting 4 constructs transformed into chemically competent *E. coli* C43 cells [56]. Each of the 4 strains was overexpressed by picking a single colony into 10 ml LB containing 100 *μ*g ml^-1^ ampicillin, growing overnight at 37°C with shaking at 200 rpm, and using this to inoculate 500 ml LB containing 100 *μ*g ml^-1^ ampicillin in a 2.5 l conical flask. This was grown for 2 hours at 200 rpm and 37°C and then induced with 0.2% (w/v) arabinose (Sigma-Aldrich, UK) and 5 *μ*g ml^-1^ all-*trans* retinal (Sigma-Aldrich, UK) and grown for a further 4 hours at 37°C. Pelleted cells were resuspended in approximately 10 ml of buffer A (25 ml K_2_HPO_4_/KH_2_PO_4_ pH 7.4) and lysed by two cycles of French pressing at a pressure of 18 000 psi. The resulting lysate was separated into two halves. Membrane fractions were isolated from one half on a 10-50% continuous sucrose gradient (the sucrose was made up in the cell resuspension buffer and 1.5 ml broken cells loaded per gradient) spun at 30,000 rpm for 2 hours at 4°C. In the second half, the detergent n-dodecyl-*β*-D-maltopyranoside (*β*-DDM) was added dropwise to a final concentration of 2% (w/v) and left at 4°C for 1 hour. The lysed cells were centrifuged at 4500×g for 20 minutes and the supernatant decanted into a fresh tube and the pellet discarded. The supernatant was applied to a self-packed anion exchange column containing DEAE-sepharose (GE Healthcare) using buffer A as running buffer. The flow-through, containing the membrane-bound proteorhodopsin complexes, was collected and centrifuged at 65000×g for 20 minutes at 4°C. The supernatant was discarded and the proteorhodopsin-containing pellet resuspended in buffer A containing 1% (w/v) of the detergent octyl-beta-glucoside (OTG) and left overnight at 4°C to solubilise the membrane-bound proteorhodopsin complexes. The solubilised membranes were centrifuged at 21000×g to remove any unsolubilised material and an absorption spectrum of the supernatant taken using an Agilent Cary 60 UV-Vis spectrophotometer.

## 3. Results

### 3.1. A Workflow to Identify and Isolate Individual CO_2_-Fixing Bacteria from Seawater

**Figure 1:**
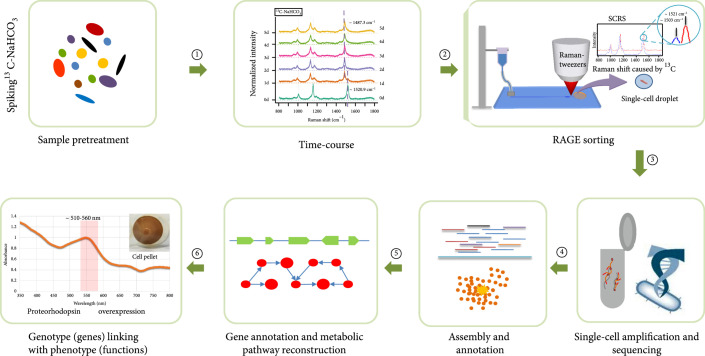
Workflow for revealing CO_2_-fixing function of bacteria in the ocean that links genotype to phenotype: ①: feeding of ^13^C-substrate to probe the metabolism of the seawater sample and then analyzing the time-course Raman spectra of carotenoids containing cells to find the shortest labeling time; ②: target cells were searched by Raman detection according to SCRS which exhibits shifts caused by ^13^C incorporation and then sorts out by the RAGE device in single-cell-single tube style; ③: the sorted cells were lysed, and its nucleic acid was amplified by MDA; then, the extracted DNA is processed for single-cell genome sequencing by high-throughput sequencing; ④: assembly and annotation for the single-cell genomic data; ⑤: metabolic functions are validated by the reconstructed metabolic pathways and genes; ⑥: proteorhodopsin overexpression was conducted for validating the PR prediction based on genomic analysis, and finally, a link between genotype (genes) and phenotype (functions) is established at the single-cell level.

It is important to link the genotype and phenotype of a single cell. This study used Raman spectra to sort cells, establishing a direct link between bacteria function and single-cell genomics (Figure 1). The seawater was sampled from a typical euphotic zone in the Yellow Sea of China (Figure S1; physiochemical parameters in Table S1). A time-course experiment was conducted to identify CO_2_-fixing bacteria in the presence of ^13^C-NaHCO_3_. Raman-activated Gravity-driven Encapsulation (RAGE) [55] was applied to sort single cells. Every single bacterial cell was sorted according to its characteristic SCRS, precisely packaged in a picoliter microdroplet, and exported in an indexed “one-cell-one-tube” manner for downstream single-cell multiple displacement amplification (MDA) and then genome sequencing (Figure 1). The single-cell genomes were reconstructed, and the key functional genes and pathways were identified, according to Raman function analysis. In this case, these include carotenoid and retinal synthesis pathway, PR synthesis, and CO_2_ fixation pathway. Finally, some novel proteorhodopsin genes were characterized and validated by expressing them in *E. coli*. Therefore, a holistic picture of the single bacterial cell in terms of function (phenotype) and genomics (genotype) is linked and revealed (Figure 1).

### 3.2. Time-Course of ^13^C Incorporation into CO_2_-Fixing Bacteria Monitored by Single-Cell Raman Spectra

**Figure 2:**
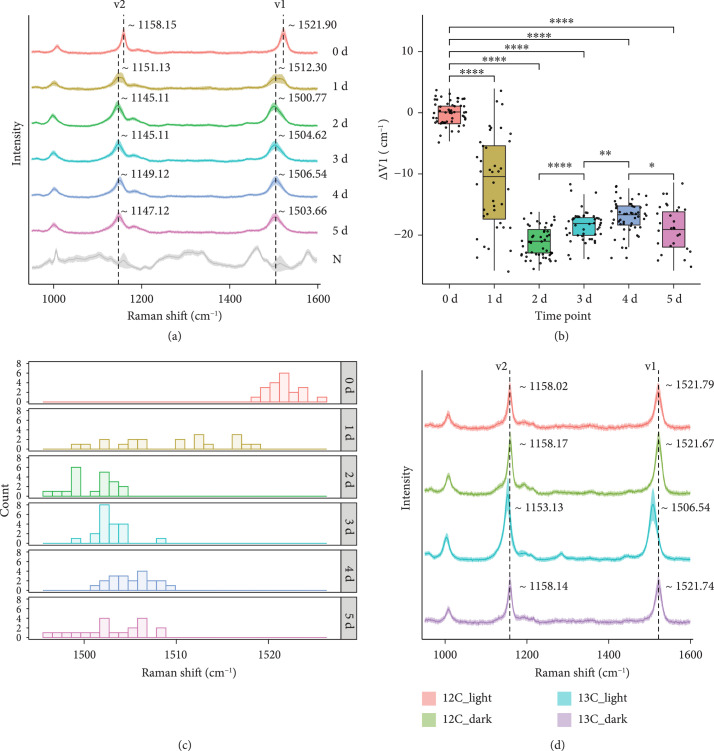
Raman spectra of carotenoid-containing cells in the seawater which were incubated in closed bottles at room temperature at different times. (a) Dynamic red shift of Raman spectra from carotenoid-containing cells in the seawater which were incubated from day 0 to day 5. Each spectrum represents an average of SCRS from ~45 single cells, and the shadow represents the standard deviation of SCRS. The Raman spectra labeled with N represent an average of SCRS from the non-carotenoid-containing cells. (b) The bar plot showed the intensity ratio of SCRS *v*1 band in each of the day groups obtained from the marine bacteria cells. (c) Histogram plots for *v*1 shift in SCRS of carotenoid-containing cells in the seawater which were incubated in closed bottles at room temperature (sampled at time points spanning from day 0 to day 5). (d) Raman spectra from carotenoid-containing cells in the seawater which were incubated with ^12^C-NaHCO_3_ or ^13^C-NaHCO_3_, under dark or normal light conditions, respectively, on day 5.

**Figure 3:**
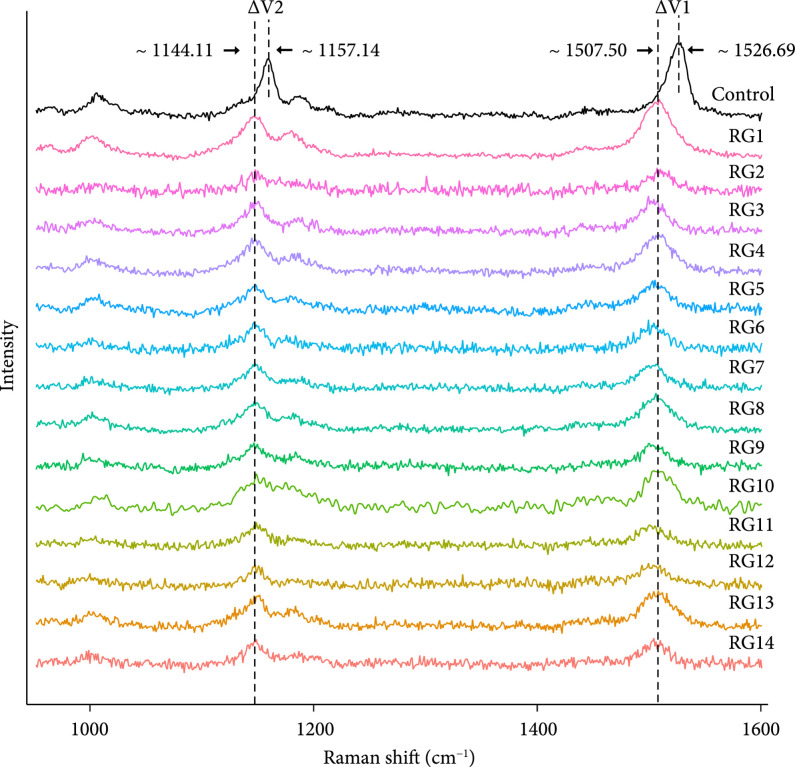
Single-cell Raman spectra of the CO_2_-fixing cells sorted by RAGE from the seawater incubated with NaHCO_3_. “Control”: ^12^C-NaHCO_3_-treated cell at 1 day of incubation; “RG1-RG14”: ^13^C-NaHCO_3_-treated cell at 1 day of incubation.

Since the integration of stable isotopes (^13^C, ^15^N, ^2^H, or ^18^O) into bacterial cells leads to significant shifts of some Raman bands in SCRS, single-cell Raman spectroscopy combined with stable isotope detection (Raman-SIP) has been used to reveal specific metabolic functions of bacteria and to screen active cells in microbial communities [10, 31, 34, 40, 57, 58]. Nearly all photosynthetic bacteria contain either carotenoids or retinoids for light harvesting and structural stabilization [15, 58]. Hence, after uptake and incorporation of ^13^C-HCO_3_- or ^13^CO_2_ into CO_2_-fixing bacteria, carotenoid Raman bands shift due to the altered vibrational energy caused by ^13^C [58]. Raman spectra of carotenoid molecules display three characteristic Raman bands (Figure S2): *v*1 (in-phase C=C), *v*2 (C–C stretching vibrations of the polyene chain), and *v*3 (in-plane rocking mode of CH_3_ groups attached to the polyene chain) [33, 58–60]. The positions of *v*1 and *v*2 in the SCRS of photosynthetic cells show stepwise shifts to lower wavenumbers when cells are incorporated with ^13^C [58]. In this study, we used the simultaneous shifts in both *v*1 and *v*2 as Raman biomarkers, to identify and sort photosynthetic cells that were actively fixing ^13^C-NaHCO_3_ in the seawater sample (Figures 2 and 3).

The time-course experiment was conducted to estimate the rate of ^13^C incorporation, and ~45 single-cell Raman spectra were measured at each time point (Figure 2(a)). Within 24 h (day 1) after incubating the seawater with ^13^C-NaHCO_3_ at room temperature, Raman shifts (due to ^13^C incorporation) in marine bacteria were detectable by Raman microspectroscopy (Figures 2(b) and 2(c)). The *v*2 bands shift from 1158 cm^-1^ to 1151 cm^-1^ while *v*1 bands shift from 1522 cm^-1^ to 1512 cm^-1^. In the following days (day 2 to day 5), the *v*2 and *v*1 bands downshifted further, to ~1145 cm^-1^ and ~1502 cm^-1^, respectively (Figure 2(a)). The data showed that after the first 24 h of incubating the seawater with NaH^13^CO_3_, Raman shifts (due to ^13^C-incorporation) among individual cells were highly heterogeneous, while at 48 h (and beyond), the degree of Raman shifts of the individual cells had all largely reached a steady state (Figures 2(b) and 2(c)).

To investigate the possibility of cross-feeding, we fed NaH^13^CO_3_ to a synthetic three-species mock microbiota that consists of *Synechococcus elongatus* PCC7942 (Se; an autotrophic bacterium), *Micrococcus luteus* OY14 (Ml; a heterotrophic bacterium), and *Saccharomyces cerevisiae* BY4742 (Sc; a heterotrophic fungus) mixed in a 1 : 1 : 1 ratio. In this mock community, after NaH^13^CO_3_ treatment for 24 h, ^13^C uptake was detected in *Synechococcus elongatus* PCC7942 (based on the “red shift” of Raman spectrum), but neither in *Micrococcus luteus* OY14 nor in *Saccharomyces cerevisiae* BY4742 (Figure S3). This observation suggests that the possibility of cross-feeding between photoautotrophic and heterotrophic bacteria within 24 h is unlikely in this study.

Based on the above results, the sampling time point for this experiment was set to day 1, and bacteria actively fixing ^13^C-NaHCO_3_ were sorted by RAGE, then analyzed by single-cell genomic sequencing (Figure 1). Dark incubation of the seawater samples in the presence of ^13^C-NaHCO_3_ was also performed and analyzed (Methods), which showed no Raman shift among the ~45 randomly selected carotenoid-containing cells under such conditions for 5 days (Figure 2(d)). Collectively, the results confirm that the Raman shift in the presence of ^13^C-NaHCO_3_ was due to light-responsive CO_2_ fixation.

### 3.3. The Impact of Bicarbonate on Microbial Community Structure in the Seawater Sample

To assess the impact of the supplemented carbon source (e.g., ^12^C-CO_2_, ^12^C-NaHCO_3_, or ^13^C-NaHCO_3_) on the microbiota structure, 16S rRNA amplicon sequencing (V3-V4) results were compared for five conditions: day 1 seawater spiked with ^13^C-NaHCO_3_ (C13-HCO3), ^12^C-NaHCO_3_ (C12-HCO3), ^12^C- (C12-CO2), ^12^C-CO_2_ plus ^12^C-NaHCO_3_ (C-combined), and the day 1 seawater control without any treatment (C-free). Three biological replicates were performed. After trimming, screening, and removal of chimeras and singletons, an average of ~64,664 high-quality reads (ranged from 52,931 to 89,432) were obtained (Table S2), showing that in this seawater sample, SAR11 bacteria (~9.6% in the community) were more abundant than cyanobacteria (~3.1%; Figure S4A). Abundance distribution suggests that the supplement of ^12^C- or ^13^C-HCO_3_^-^ had introduced minor impacts on the structure of the microbial community, compared to the original seawater control without any supplement (Figure S4A). Moreover, principal coordinate analysis (PCoA) and UPGMA clustering analysis were performed to evaluate the similarity in the microbial community structure of all samples using the weighted UniFrac distance, which incorporates the degree of divergence in the phylogenetic tree of operational taxonomic units (OTUs). Both analyses show that the C-combined and ^12^C-CO_2_ samples were quite similar to each other (probably due to the CO_2_-caused acidification of seawater), while the HCO_3_^-^-adding groups were more similar to the C-free group (Figures S4B and S4C). Therefore, since sodium bicarbonate as an external carbon source has a minimal impact on the structure of the seawater microbiota, ^13^C-HCO_3_^-^ probing, which was the option we chose here, should more precisely model the in situ functional activity in the seawater sample, than other ^13^C-labeled carbon substrates tested above.

### 3.4. Linking CO_2_ Fixation Functions to the Underlying Genome at Single-Cell Resolution

After one-day incubation of seawater with ^13^C-NaHCO_3_, cells were sorted using RAGE (Figure 1; Methods). Cells whose SCRS display both carotenoid bands and ^13^C shifts were sorted by RAGE (Figures 1 and 3) according to the Raman sorting criteria (Figure 2). The sorted cells trapped in the droplets were transferred into PCR tubes, with each tube containing only one single cell.

Following labeling with ^13^C-NaHCO_3_ for 24 hours, 14 individual bacterial cells were sorted independently from the same seawater sample using the sorting criteria (Figures 2 and 3). All sorted cells showed significant Raman shifts in the *v*1 and *v*2 bands in SCRS (Figure 3), indicating a likely CO_2_-fixing function. In order to correlate the observed phenotype (e.g., CO_2_ fixation) via Raman analysis and the genotype at the single-cell level, the sorted cells were processed to do single-cell genomic analysis. Cells were initially lysed, and then, the lysates were used as templates for MDA. The 16S rRNA sequence of MDA products was examined by PCR to confirm successful amplification (Figure S5; primers shown in Table S3).

**Figure 4:**
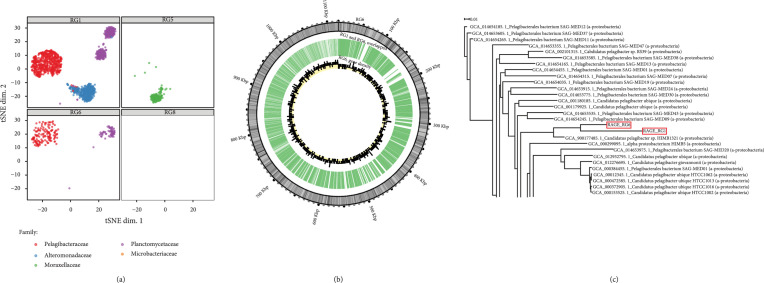
Binning of SAGs from single-cell genomes derived from one-cell RAGE-Seq from seawater samples. (a) The t-SNE projection of binned contigs from post-RACS single-cell sequencing reveals their taxonomical origin. Contigs are visualized based on 4-mer frequency features. Samples are marked with distinct shapes. Each contig is colored based on its taxonomic annotation at the family level. (b) Circos plot representation of overlaps between recovered *Pelagibacter* spp. genomes from RG1 and RG6. The RG6 genome is linked from all contigs and represented as the outer track, with coordinates marked on the outermost circle and GC content as an inner heat map. Overlap regions between contigs of RG1 and RG6 are shown as the middle track. The gene numbers per kilobase for RG6 are given as the innermost track. Overall, ~71.25% genomes of RG6 (in length) also have evidence in RG1. (c) Phylogenetic tree based on available *Pelagibacteraceae* genomes (partial; the complete figure in Figure S5). RG1 and RG6 are compared with genomic sequences of 89 *Pelagibacteraceae* obtained from NCBI GenBank (GCA) assembly. All genomes were annotated using Prokka. STRIDE was used by OrthoFinder [83] for inferring the root of a species tree by analyzing the distribution of gene duplication events in the genomes of the species in the tree (http://www.stevekellylab.com/stride).

In the same batch of experiments, 6 out of 14 one-cell samples had positive results in the 16S rRNA PCR test. Six positive samples were numbered RG1, RG5, RG6, RG8, RG9, and RG11 (Figure S5). Among these MDA-positive samples, high-throughput sequencing libraries of four (RG1, RG5, RG6, and RG8) were successfully constructed and sequenced (Methods). After quality control, ~17.4 million clean reads for each sample were obtained for data assembly (Table S4). To assign the taxonomy of the target cell, we have employed both GTDB and BLASTN for the RAGE-Seq-derived one-cell assemblies, which revealed essentially identical results (Table S5). According to de novo genome assembly and homology-based annotation, the sorted bacteria belong to five phyla: *Pelagibacteraceae*, *Moraxellaceae*, *Microbacteriaceae*, *Planctomycetaceae*, and *Alteromonadaceae* (Table 1). Then, assembled contigs (>2,000 bp), which represented the clusters of genomes in each sample, were visualized using t-SNE (Figure 4(a)).

**Table 1:** Predicted genome completeness and 16S rRNA genes of RAGE-sorted bacteria.

RAGE-sorted samples	Taxonomic classification	Estimated genome completeness by CheckM (%)	Identity score for the identified 16S rRNA in SCGs (%)	Abundance in the community (C-free/^13^C-HCO_3_)	Reference supporting presence of carotenoids	Reference evidence of carbon dioxide fixation
RG1	*Pelagibacteraceae*	97.6	99.6	7.65%/5.84%	[78]	—
	*Planctomycetaceae*	74.5	—	—	—	—
	*Alteromonadaceae*	72.9	—	0/0	—	—

RG5	*Moraxellaceae*	76.4	99.7	0/0	[79]	[80]

RG6	*Pelagibacteraceae*	80.0	99.6	7.65%/5.84%	[78]	—
	*Planctomycetaceae*	24.4	—	—	—	—

RG8	*Microbacteriaceae*	10.7	100.00	6.49%/7.84%	[81]	[82]

The dominant contigs in RG1 and RG6 were assigned to the SAR11 bacteria of *Pelagibacteraceae*, although a small portion of contigs was assigned to *Planctomycetaceae* and *Alteromonadaceae* (Figure 4(a), Table 1, and File S1). The cross assignment might be due to either the previously unknown *Pelagibacteraceae*, DNA contaminations, or other symbiotic bacteria. The >1 kb PCR products of 16S rRNA genes (27F and 1492R) from MDA of RG1 and RG6 were purified and sequenced, which showed that both are >99% identical to “*Candidatus* Pelagibacter ubique” HTCC1062 [61] (File S2).

Based on the RAGE sorting criteria, the targeted cells should contain shifted carotenoid bands in their SCRS due to the incorporation of ^13^C from ^13^C-NaHCO_3_. According to the literature, the sorted bacteria in the three families *Pelagibacteraceae*, *Moraxellaceae*, and *Microbacteriaceae* are able to synthesise carotenoids (Table 1), consistent with the sorting criteria. Although it has been reported that SAR11 can employ PR to harvest sunlight as an energy source [5], it was not known whether SAR11 bacteria could use light to drive the assimilation of HCO_3_^-^ in the natural environment.

According to the Raman sorting criteria, the sorted *Pelagibacter* spp. should contain carotenoids and also have the ability to incorporate ^13^C-NaHCO_3_. *Pelagibacter* spp. was in the first group of bacteria (day 1) found to be labeled with ^13^C in the seawater sample, which was taken from the euphotic zone with a low level of organics (chemical oxygen demand 1.7 mg/l, Table S1). These data suggest that *Pelagibacter* spp. should have both light-utilizing machinery and CO_2_ fixation ability. Hence, it is predicted that we should find genes encoding *β*-carotene biosynthesis, 15,15′-dioxygenase, proteorhodopsin (PR), and CO_2_ fixation pathway in the reconstructed single-cell genome of the sorted *Pelagibacter* spp. Remarkably, our whole genome analysis indeed found most of these genes in the sorted *Pelagibacter* spp., as discussed below.

### 3.5. Reconstruction of the *β*-Carotene Biosynthesis Pathway from Single-Cell Genomic Data

For the reconstructed *Pelagibacter* spp. genome of RG1 and RG6, GC content was 29.3% and 29.2%, respectively (Table 2), consistent with the 89 sequenced *Pelagibacter* spp. strains in the NCBI database (27.8% to 33.4%, with an average of 29.5%). Their completeness was 97.6% and 80.0%, respectively, as estimated by CheckM (based on 695 marker genes from 15 *Pelagibacteraceae genome*s; Table 1), indicating that RAGE was able to obtain high genome coverage of just one bacteria cell from the microbial community (Figure 1). The reconstructed *Pelagibacter* spp. genomes of RG1 and RG6 revealed the sharing of 997 kb, i.e., 71.2% genomic content of the latter (Figure 4(b)). A phylogenetic tree of various sequenced SAR11 genomes confirms the identity of the reconstructed genomes from RG1 and RG6 as *Pelagibacter* spp. (Figure 4(c) and Figure S6).

**Table 2:** Data statistics of *Pelagibacterales* obtained from the functional single-cell genomes of samples RG1 and RG6.

Sample	*Pelagibacter* spp. in RG1	*Pelagibacter* spp. in RG6
Contig number	5,400	862
Bases	5.036 Mbp	1.111 Mbp
N50	1,862	3,019
GC%	29.3	29.2
Gene	4,998	1,144

**Figure 5:**
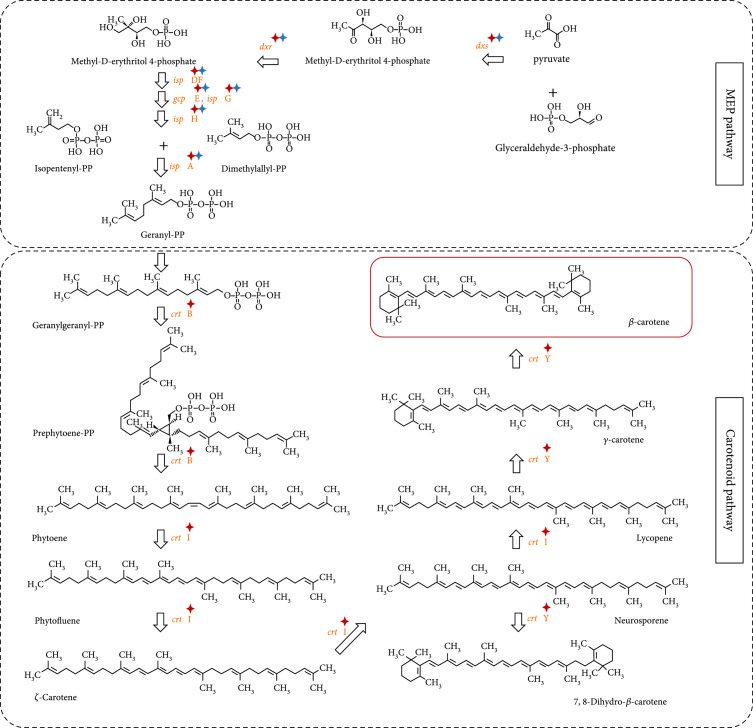
Reconstructed *β*-carotene module in the carotenoid synthesis pathway from the single-cell *Pelagibacter* spp. genomes derived by RAGE-Seq. The known pathways (used as reference) were obtained from the KEGG database. Enzyme genes with red asterisk: found in the *Pelagibacter* spp. from RG1; with blue asterisk: found in the *Pelagibacter* spp. from RG6.

The complete gene set for carotenoid biosynthesis was identified in the reconstructed low-GC-content *Pelagibacter* spp. contigs of RG1 and RG6 but absent in other parts of sequences in the corresponding MDA products (Figure 5 and Table 1). As ^13^C shift of carotenoid Raman bands was used as criteria for the cell sorting (Figure 3), the SAR11 bacteria of *Pelagibacter* spp. must be the targeted bacteria that contained carotenoids and incorporated ^13^C from ^13^C-HCO_3_ in the seawater (Table 1 and Figure 4(a)).

**Figure 6:**
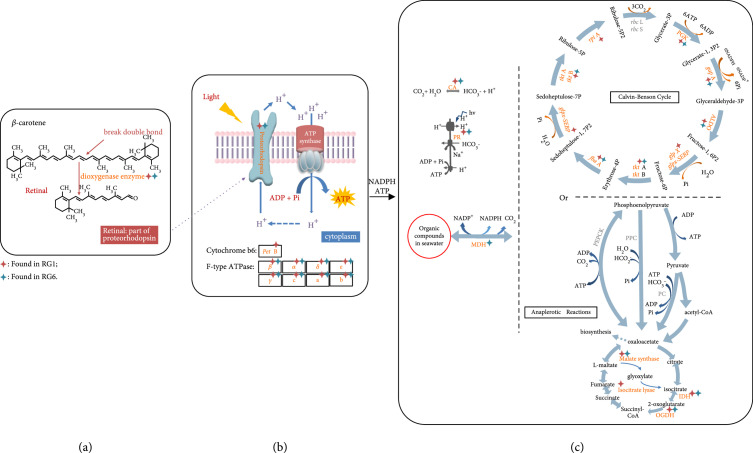
Reconstructed CO_2_-fixing pathways from the single-cell *Pelagibacter* spp. genomes derived by RAGE-Seq. (a) *β*-carotene can be cleaved by dioxygenase into retinal, which is an important element in functional PR; (b) light-harvesting PR; (c) reconstruction of Calvin-Benson cycle pathway and the anaplerotic reactions. The known pathways were obtained from the KEGG database. Enzyme genes with red asterisk: found in the *Pelagibacter* spp. from RG1; with blue asterisk: found in the *Pelagibacter* spp. from RG6. CA: carbonic anhydrase; PR: proteorhodopsin; PEPCK: phosphoenolpyruvate carboxykinase; PPC: phosphoenolpyruvate carboxylase; PC: pyruvate carboxylase; MDH: malate dehydrogenase; IDH: isocitrate dehydrogenase; OGDH: 2-oxoglutarate dehydrogenase.

To reveal the carbon fixing potential of these functional single-cell genomes, the two single-cell gene sets from the *Pelagibacter* spp. were mapped to the KEGG pathways, which predict gene function mainly based on sequence homology [62]. Within these gene sets, seven metabolic pathways were examined, including ko00195 (photosynthesis), ko00710 (carbon fixation in photosynthetic organisms), ko00906 (carotenoid biosynthesis), ko00720 (carbon fixation pathways in prokaryotes), ko00860 (porphyrin and chlorophyll metabolism), ko00830 (retinol metabolism), and ko00900 (terpenoid backbone biosynthesis). Most of the seven pathways were detected in these four single-cell genomic samples in our study (details in File S3). Genes encoding the pathway for the synthesis of *β*-carotene synthesis were identified in the reconstructed *Pelagibacter* spp. genomes from RG1 and RG6 samples (Figure 5), consistent with the RAGE sorting criteria. *Pelagibacter* genome obtained from RG1 has all the genes for carotenoid synthesis, and only a partial of these genes were found in RG6 (the specific links between the genomes and the genes are highlighted in Figures 5 and 6).

### 3.6. Establishment of a Light-Utilizing System and a CO_2_-Fixing Pathway in the RAGE-Seq-Derived SAR11 Bacteria

*β*-Carotene is the precursor of retinal, a key component of functional PR (Figure 5); the 15,15′-dioxygenase cleaves one molecule of *β*-carotene into two molecules of retinal, which is the cofactor binding within PR to make functional holo-PR (Figure 6(a)) [15, 63]. We not only found the pathway for *β*-carotene biosynthesis but also identified 15,15′-dioxygenase genes from the single-cell *Pelagibacter* spp. genomes from both RG1 and RG6 samples (Figure 6(a), Figure S7A, and File S4). Notably, we also identified four other proteorhodopsin (PR) genes from single-cell genomes RG1 and RG6 (Figure 6(b), Figures S7B and S8, and File S5). In contrast, the genes required for chlorophyll-based photosynthesis, encoding the biosynthesis of chlorophyll *a*, the phycobilisome, and the biogenesis of Photosystem I and Photosystem II complexes [64] were all absent from the genome, demonstrating the absence of this chlorophyll mode of photosynthesis from the reconstructed *Pelagibacter* spp. genomes. Collectively, we have identified the genetic machinery to make holo-PR, and the presence of additional genes encoding the F-type ATPase (Figure 6(b)) suggests that *Pelagibacter* spp. is able to form a complete “photon to ATP” loop, consistent with the observation in the pure culture of SAR11 strain HTCC1062 [30].

**Figure 7:**
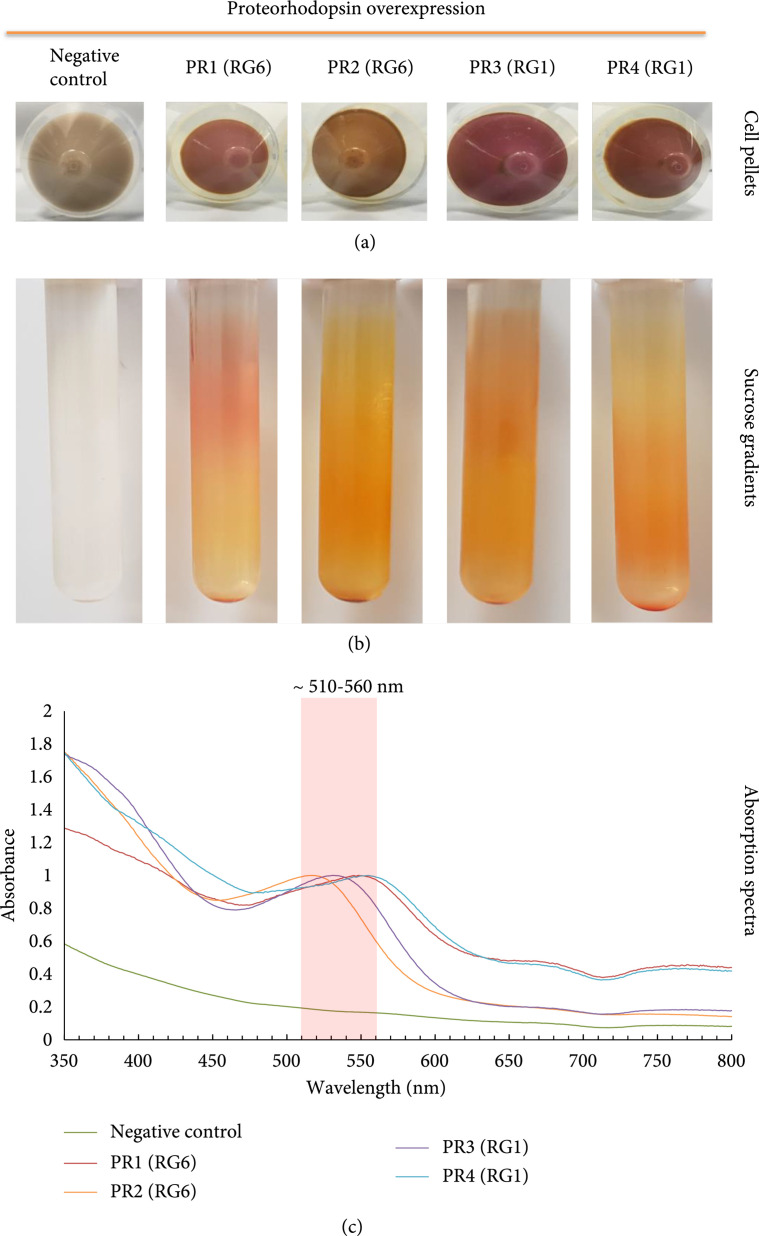
Functional characteristics of the PRs mined from the single-cell *Pelagibacter* spp. genomes derived by RAGE-Seq. (a) Cell pellets obtained after induction with arabinose and retinal for four *E. coli* C43 strains harbouring pBAD constructs that contain the putative proteorhodopsin (PR) genes from SCG data of RG1 and RG6, respectively. A pellet from the uninduced C43 strain containing pBAD:Hoch PR is shown as the negative control for comparison. (b) Separation of cell membranes from lysed cell pellets following their application to 10-50% continuous sucrose gradients and centrifugation. (c) Absorption spectra of the detergent-solubilised cell membrane pellets that contain the purified PR.

In order to validate the PR prediction based on genomic analysis of RG1 and RG6, we synthesised six PR fragments according to the sequencing data shown in File S5. These putative PR genes were cloned into plasmids and transferred into *E. coli* C43 strain, which is able to overexpress intracellular membranes for hosting membrane protein PR [56]. The expression of PRs was induced by 0.2% (w/v) arabinose in a LB medium supplemented with all-*trans* retinal. The negative control is *E. coli* C43 with plasmid backbone that did not produce any pigment, while four of six putative PR genes (PR1-4) cloned into *E. coli* C43 strains show clear pigment production (Figure 7(a)). The purified membrane of these four clones contains pigments (Figure 7(b)), and the holo-PRs absorb light between 510 and 560 nm (Figure 7(c)), which are the typical absorption wavelength of PR. Among these four PR genes (File S5), PR2 has 98% homology to a PR gene in *Candidatus Pelagibacter* sp. HIMB1321 (GenBank: LT840186.1), and PR3 has 94% homology to alpha proteobacterium HIMB59, an unclassified *Pelagibacteraceae* (GenBank: CP003801.1). Interestingly, PR1 and PR4 have no significant homology to any genes in GenBank, NCBI. However, the translated peptide sequence of PR1 is of 90% homology to xanthorhodopsin and 80% homology to bacteriorhodopsin, and PR4 is of 59% homology to rhodopsin in *Planctomycetia bacterium*. Hence, PR1 and PR4 are novel proteorhodopsin genes and successfully expressed and validated in *E. coli*.

According to the sequencing and binning results, two possible pathways (Calvin-Benson cycle pathway and the anaplerotic reactions) to fix CO_2_ are shown in Figure 6(c). Most of the genes, except *rbcL* and *rbcS*, involved in the Calvin-Benson cycle of CO_2_ fixation have been identified in the reconstructed single-cell genomes from the sorted RG1 and RG6 *Pelagibacter* spp. samples (Figure 6(c)). However, some essential genes encoding enzymes catalysing from phosphoenolpyruvate to oxaloacetate in the anaplerotic reactions were absent (Figure 6(c)). These data suggest that the PR-containing *Pelagibacter* spp. is likely to have the genetic machinery to encode the Calvin-Benson cycle for CO_2_ fixation (Figure 6(c)) in the sea.

In summary, we used SCRS to identify bacterial cells that contain carotenoids and fix CO_2_, based on ^13^C-NaHCO_3_ labeling (Figure 3), which were then sorted, encapsulated, and whole-genome sequenced at the precisely one-cell resolution, creating a link for each cell between its physiological properties and its genetic composition. The reconstructed single-cell genomes of the SAR11 bacteria in the sample identified individual cells of *Pelagibacter* spp. harbouring the genes for synthesis of carotenoids (Figure 5), including retinal, proteorhodopsin, enzymes of CO_2_ fixation, and the F-type ATPase (Figure 6). Thus, each cell has the genetic information for fixing CO_2_, driven by the solar energy harvested by PR, and therefore, *Pelagibacter* spp. could contribute to carbon cycling in the oceans.

## 4. Discussion

### 4.1. Novel Raman-Activated Cell Sorting to Link Bacterial Functions and Single-Cell Genomics

The marine microbiome constitutes the bulk of the biomass in the ocean, and knowledge of its composition and physiological properties is fundamental for a deep scientific understanding of the global carbon/nitrogen cycle, the solar energy budget, and sustainable exploitation of marine resources. However, most bacteria in the marine microbiome remain uncultured [2, 3]. Thus, there is a need to develop methods for probing the function of environmental microbiomes in situ, which are driven by the following requirements: (i) dissection of the microbiota structure by sequencing and tracking microbiota state, function, and intercellular interaction and (ii) interrogation of a consortium or population of bacteria and the ability to probe individual bacterial cells. Many studies have successfully demonstrated that Raman-SIP is able to precisely pinpoint bacteria with specific metabolic functions at the single-cell level [10, 31, 36, 38, 39, 42, 43, 46, 49, 52, 65]. However, it is important to establish links between metabolism and genomes at the single-cell level using RACS [32, 33].

Although RACE has been developed to sort single cells and sequence their genomes according to bacterial SCRS, genome coverage for single cells was low [36]. Various attempts have been made to improve the genomic quality for single-cell sequencing [66, 67], but high coverage of single-cell genomes remains challenging. Technical limitations, such as high amplification bias with very few DNA templates, lead to lower success rates in single-cell genome amplification and lower target gene hit rates in sequencing data analysis [68]. In previous work, we attempted the identification of CO_2_-fixing bacteria in the Yellow Sea, but the difficulties in obtaining single-cell genomics required the pooling of over 30 cells for “minimetagenome sequencing” [10].

To tackle these challenges, we applied the RAGE to the marine sample, which sorts and encapsulates precisely one cell on the basis of its SCRS and minimises photo- and thermal damage to single cells by operating in water [55]. More importantly, picoliter encapsulation by RAGE can be readily transformed into an emulsion system, which effectively reduces the MDA reaction volume and achieves high-coverage sequencing of a one-cell genome. We showed that, for marine microbiome samples, SCRS are sufficiently sensitive to identify candidate cells actively fixing CO_2_ and that for each such cell RAGE allows precisely one-bacterial-cell-per-reaction MDA reaching almost 98% sequence coverage. The ability to profile and correlate bacterial metabolism and high-quality genome sequences at one-cell resolution illustrates the potential for a broad application of such Raman-activated cell sorting and sequencing approaches (RACS-Seq; for a detailed comparison of the various RACS-Seq methods, please refer to Table S2 from [34]).

Notably, the assembled genome RG1 was unusually large (~5 Mbp), much larger than the typical size of *Pelagibacter* spp. genomes and the ~1.1 Mbp size of *Pelagibacter* spp. from RG6 (Table 2), although the *Pelagibacter* spp. from RG1 and RG6, respectively, share 71.2% genomic content. It is possible that RG1 might contain either contaminated DNA or other symbiotic bacteria, although contamination of single-cell sequencing by free floating or cell-surface-attached environmental DNA is usually hard to avoid in field samples [69–74]. However, RG1 and RG6 were identified as SAR11 bacteria with the following evidence: (i) the nearly full-length 16S rRNA sequences (>1.0 kb) of PCR products from MDA of RG1 and RG6 indicated both as *Pelagibacter* spp. (File S2). (ii) Per CheckM, completeness of the *Pelagibacter* spp. genome was 97.6% for RG1 and 80.0% for RG6 (Table 1). (iii) The binning result shows that the GC contents of RG1 (~5 Mbp) and RG6 (~1.1 Mbp) were 29.3% and 29.2%, consistent with the typical SAR11 genome feature of low GC content. (iv) Key functional gene sets including carotenoid synthesis, dioxygenase, and PR were found in the reconstructed genomes of *Pelagibacter* spp. in RG1 and RG6 (Figure 6) but absent in those non-*Pelagibacter* spp. contigs (e.g., *Planctomycetaceae* and *Alteromonadaceae* contigs) in RG1 and RG6.

In the one-cell RAGE-Seq reactions of mock microbiota, without any exception, the top contig bin from each of such precisely one-cell assemblies corresponds to the target cell (i.e., the single-cell 16S rRNA sequencing-based genotype is always consistent with the SCRS-predicted phenotype such as whether the cell harbours pigments), supporting the low possibility of contamination in the RAGE-Seq workflow [34]. The presence of both *Pelagibacteraceae* and *Planctomycetaceae* in RG1 and RG6 might implicate the symbiosis hypothesis. However, this hypothesis does not change the key conclusion of this study, i.e., *Pelagibacter* spp. possesses most of the genes encoding enzymes for CO_2_ fixation and the full genes necessary for carotenoid synthesis. It is however beyond the scope of this study to validate the symbiosis hypothesis.

### 4.2. SAR11 in the Oceans Can Fix CO_2_ in the Presence of Light and Its Implication

In this study, we applied RAGE to obtain single-cell genomes from CO_2_-fixing bacteria identified by SCRS. We reconstructed genomes of CO_2_-fixing bacteria originating from the Yellow Sea in China, which were able to assimilate ^13^C-HCO_3_^-^ as short as 24 hours at room temperature under natural light, while no CO_2_-fixating bacterium was observed in the dark experiments. The MDA amplified genomic DNA of the isolated single cells were checked by PCR using 27F and 1492 primers (Table S3), which cover nearly the full 16S rRNA gene length. A typical SAR11 bacterium *Pelagibacter* spp. was found as the single species in RG1 and RG6 MDA samples. Moreover, GC contents of RG1 and RG6 were 29.3% and 29.2%, respectively, both very similar to those of the other 89 sequenced *Pelagibacter* spp. strains in the NCBI database (27.8% to 33.4%, with an average of 29.5%). Single-cell genomics revealed that this *Pelagibacter* spp. contains an entire rhodopsin-based system, including four genes encoding PR and its retinal cofactor. The PR genes were validated by cloning and expression of these genes in *E. coli.* Thus, we have linked phenotype (the presence of carotenoids), function (the incorporation of ^13^C-HCO_3_^-^), and genotype for a series of *Pelagibacter* spp. cells, which we suggest as self-sufficient for light-powered energy conversion in the oceans.

The SAR11 clade of marine bacteria has an estimated global population of $2.4\times 10^{28}$ cells, and conventional metagenomic sequencing indicates that global SAR11 bacteria account for an estimated 25% of all plankton [29, 30]. They are typical oligotrophic microorganisms and are the most abundant bacteria in ocean surface waters, but they reach their maximum number in stratified oligotrophic gyres, which are an expanding habitat in the warming oceans [29]. SAR11 bacteria reportedly play an important role in ocean carbon and nutrient cycling [75, 76]. They are dominant in the ocean and usually contain PR, and a recent survey indicates the significant contribution of microbial rhodopsins to solar energy harvesting in the oceans [6, 29].

Although SAR11 is widely distributed and abundant in the world, our understanding of its ecological effects is limited due to its difficulty in cultivation and the lack of appropriate research tools. We sampled seawater from a euphotic and oligotrophic zone, which has a relatively warm temperature of 16.9°C, plenty of light, and very low chemical oxygen demand (1.7 mg/l) (Table S1), conditions favoring bacteria that are able to harvest light and fix CO_2_. It is likely that some SAR11 bacteria use organic compounds as electron donors and ATP from PR-light mediated proton pump to fix CO_2_ (Figure 6). In this model, organic compounds only serve as electron donors, and the energy is provided by PR-based light harvesting, so low energy organics would be sufficient to support cell growth. Indeed, we have shown formate was able to support the growth of PR expressing bacteria (containing genes for the Calvin-Benson cycle) in a separate research [77]. We have used Raman-based imaging, labeling, and sequencing methods to establish a link between PR content and CO_2_ fixation at the level of single cells, for the SAR11 bacterium *Pelagibacter* spp. In summary, this study demonstrates that RAGE-mediated analysis of a single-cell genome can establish a reliable link between the phenotype and genotype of uncultured bacteria in the ocean, which solves a basic problem and paves the way for function-based dissection of the “biological dark matter” in the environment.

## Data Availability

The sequence data reported in this paper have been deposited to the NCBI SRA database with bioproject accession PRJNA609879 and PRJNA611094.

## Supplementary Materials

Supplementary Materials Supplementary file 1: identified 16S rRNA genes from single-cell Pelagibacter spp. genomes from RG1 and RG6. Supplementary file 2: identified 16S rRNA genes obtained from MDA products of SAGs from the RAGE-Seq samples. Supplementary file 3: list of carbon metabolism-related genes and their annotations in the single-cell Pelagibacter spp. genomes from RG1 and RG6. Supplementary file 4: identified beta-carotene 15,15′-dioxygenase genes from the single-cell Pelagibacter spp. genomes from RG1 and RG6. Supplementary file 5: identified proteorhodopsin (PR) genes from the single-cell Pelagibacter spp. genomes from RG1 and RG6.
