# Supplementary material for: Revealing CO2-Fixing SAR11 Bacteria in the Ocean by Raman-Based Single-Cell Metabolic Profiling and Genomics
Source: Biodes Res. 2022 Oct 13;2022:9782712. doi: 10.34133/2022/9782712 (PMC10521720; doi:10.34133/2022/9782712)
Supplement: Supplementary Materials — Supplementary file 1: identified 16S rRNA genes from single-cell Pelagibacter spp. genomes from RG1 and RG6. Supplementary file 2: identified 16S rRNA genes obtained from MDA products of SAGs from the RAGE-Seq samples. Supplementary file 3: list of carbon metabolism-related genes and their annotations in the single-cell Pelagibacter spp. genomes from RG1 and RG6. Supplementary file 4: identified beta-carotene 15,15′-dioxygenase genes from the single-cell Pelagibacter spp. genomes from RG1 and RG6. Supplementary file 5: identified proteorhodopsin (PR) genes from the single-cell Pelagibacter spp. genomes from RG1 and RG6. [file 9782712.f1.zip › Supplementary file1-file 5.pdf]

Supplementary file 1. Identified 16S rRNA genes from single-cell *Pelagibacter* spp. genomes from RG1 and RG6.

>RG1_DGDANKKC_14321 16S ribosomal RNA (partial) 16S ribosomal RNA

AAGGTCCGGCTAACTTCGTGCCAGCAGCCGCGGTAATACGAAGGGACCTAGCGTAGTTC
GGAATTACTGGGCTTAAAGAGTTCGTAGGTGGTTGAAAAAGTTAGTGGTGAAATCCCAG
AGCTTAACTCTGGAACTGCCATTAAAACTTTTCAGCTAGAGTATGATAGAGGAAAGCAG
AATTTCTAGTGTAGAGGTGAAATTCGTAGATATTAGAAAGAATACCAATTGCGAAGGCA
GCTTTCTGGATCATTACTGACACTGAGGAACGAAAGCATGGGTAGCGAAGAGGATTAG
ATACCCTCGTAGTCCATGCCGTAAACGATGTGTGTTAGACGTTGGAAATTTATTTTCAGT
GTCGCAGCGAAAGCGATAAACACACCGCCTGGGGAGTACGACCGCAAGGTTAAAACTC
AAATGAATTGACGGGGACCCGCACAAGTAGTGGAGCATGTGGTTTAATTCGAAGATAC
GCGCAGAACCTTACCAACACTTGACATGTTCGTCGCGACTCTAAGAGATTAGAGTTTTC
GGTTCGGCCGGACGAAACACAGGTGCTGCATGGCTGTCGTCAGCTCGTGTCGTGAGATG
TTGGGTTAAGTCCCGCAACGAGCGCAACCCTCACTTTTAGTTGCCATCATTAAGTTGGGC
ACTCTGAAAGAACTGCCAGTGATAAGCTGGAGGAAGGTGGGGATGACGTCAAGTCCTC
ATGGCCCTTACGTGTTGGGCTACACACGTGCTACAATGGTATCTACAACAGGAAGCAAG
ACTGCGAGGTCAAGCAAATCCTTAAAAGATACCTCAGTTCGGATTGCACTCTGCAACTC
GAGTGCATGAAGCTGGAATTACTAGTAATCGTGGATCAGCGTGCCACGGTGAATGCGTT
CCCGGGTCTTGTACACACCGCCCGTCACACCATGGGAGTTGGTTCTACCTTAAGGCAAG
GTTTCAAACCCTTGACCACGGTATAGTCAGCGACTAGGGTGAAGTCGTAACAAGGTAGC
CGTAGGGGAACCTGCGGCTGGATTACCTCCTT

>RG1_DGDANKKC_29682 16S ribosomal RNA (partial) 16S ribosomal RNA

AGGTCCGGCTAACTTCGTGCCAGCAGCCGCGGTAATACGAAGGGACCTAGCGTAATTCG
GAATTACTGGGCGTAAAGCGCGCGTAGGCGGTTAAGTAAGTTAATTGTGAAAGCCCAA
AGCTCAACTTTGGAATTGCAATTAAAACTACTTAGCTAGAGTTTATCAGAGGAAAGCGG
AATACATAGTGTAGAGGTGAAATTCGTAGATATTATGTAGAACACCAGTTGCGAAGGCG
GCTTTCTGGGATAACACTGACGCTGAGGTGCGAAAGTATGGGTAGCGAAGAGGATTAG
ATACCCTCGTAGTCCATACCGTAAACGATGATTGTTAGATGTTGGAAATTTATTTTCAGT

ATCACAGCTAACGCGTTAAACAATCCGCCTGGGGAGTACGACCGCAAGGTTAAAACTCA
AATGAATTGACGGGGACCCG

>RG6_HJAEHAFH_02553 16S ribosomal RNA

TTGAGAGTTTGATCATGGCTCAGAACGTACGCTGGCGGCACGCCTAACACATGCAAGTC
GAACGAAGTAGCAATACTTAGTGGCAGACGGGTGAGTAACATGTGGGTATCTGCCCTTT
GGCCTGGAATAACACGAGGAAACTTGTGCTAATACCGGATAAGTCTTTACGGAGAAAG
CTTTATGCACCATTGGATGAGCCCGCACTTGATTAGTTTGTTGGTGGGGTAATGGCCTAC
CAAGACTGTGATCAATAGCTGATTTGAGAGGATGATCAGCCACATTGGGACTGAGACAC
GGCCCAAACTCCTACGGGAGGCAGCAGTGGGGAAACTCTGATGCAGCGATGCCGCGTG
AGTGAAGAAGGCCCTTGGGTTGTAAAGCTCTTTCGTCGGGGAAGAAAATGACTGTACCC
GAATAAGAAGGTCCGGCTAACTTCGTGCCAGCAGCCGCGGTAATACGAAGGGACCTAG
CGTAGTTCGGAATTACTGGGCTTAAAGAGTTCGTAGGTGGTTGAAAAAGTTAGTGGTGA
AATCC

>RG6_HJAEHAFH_04729 16S ribosomal RNA

TCGTCAGCTCGTGTCGTGAGATGTTGGGTTAAGTCCCGCAACGAGCGCAACCCTCACTTT
TAGTTGCCATCATTAAGTTGGGCACTCTGAAAGAACTGCCAGTGATAAGCTGGAGGAAG
GTGGGGATGACGTCAAGTCCTCATGGCCCTTACGTGTTGGGCTACACACGTGCTACAAT
GGTATCTACAACAGGAAGCAAGACTGCGAGGTCAAGCAAATCCTTAAAAGATACCTCA
GTTCGGATTGCACTCTGCAACTCGAGTGCATGAAGCTGGAATTACTAGTAATCGTGGAT
CAGCGTGCCACGGTGAATGCGTTCCCGGGTCTTGTACACACCGCCCGTCACACCATGGG
AGTTGGTTCTACCTTAAGGCAAGGTTTCAAACCCTTGACCACGGTATAGTCAGCGACTG
GGGTGAAGTCGTAACAAGGTAGCCGTAGGGGAACCTGCGGCTGGATTATCTCCTT

Supplementary file 2. Identified 16S rRNA genes obtained from MDA products of SAGs from the RAGE-Seq samples.

>RG1_ 16S ribosomal RNA gene

GGTAATCCAGCCGCAGGTTCCCCTACGGCTACCTTGTTACGACTTCACCCTAGTCGCTGA
CTATACCGTGGTCAAGGGTTTGAAACCTTGCCTTAAGGTAGAACCAACTCCCATGGTGT
GACGGGCGGTGTGTACAAGACCCGGGAACGCATTCACCGTGGCACGCTGATCCACGATT
ACTAGTAATTCCAGCTTCATGCACTCGAGTTGCAGAGTGCAATCCGAACTGAGGTATCT
TTTAAGGATTTGCTTGACCTCGCAGTCTTGCTTCCTGTTGTAGATACCATTGTAGCACGT
GTGTAGCCCAACACGTAAGGGCCATGAGGACTTGACGTCATCCCCACCTTCCTCCAGCT
TATCACTGGCAGTTCTTTCAGAGTGCCCAACTTAATGATGGCAACTAAAAGTGAGGGTT
GCGCTCGTTGCGGGACTTAACCCAACATCTCACGACACGAGCTGACGACAGCCATGCAG
CACCTGTGTTTCGTCCGGCCGAACCGAAAACTCTAATCTCTTAGAGTCGCGACGAACAT
GTCAAGTGTTGGTAAGGTTCTGCGCGTATCTTCGAATTAAACCACATGCTCCACTACTTG
TGCGGGTCCCCGTCAATTCATTTGAGTTTTAACCTTGCGGTCGTACTCCCCAGGCGGTGT
GTTTATCGCTTTCGCTGCGACACTGAAAATAAATTTCCAACGTCTAACACACATCGTTTA
CGGCATGGACTACGAGGGTATCTAATCCTCTTCGCTACCCATGCTTTCGTTCCTCAGTGT
CAGTAATGATCCAGAAAGCTGCCTTCGCAATTGGTATTCTTTCTAATATCTACGAATTTC
ACCTCTACACTAGAAATTCTGCTTTCCTCTATCATACTCTAGCTGAAAAGTTTTAATGGC
AGTTCCAGAGTTAAGCTCTGGGATTTCACCACTAACTTTTTCAACCACCTACGAACTCTT
TAAGCCCAGTAATTCCGAACTACGCTAGGTCCCTTCGTATTACCGCGGCTGCTGGCACG
AAGTTAGCCGGACCTT

>RG5_ 16S ribosomal RNA gene

GAGTTTGATCATGGCTCAGATTGAACGCTGGCGGCAGGCTTAACACATGCAAGTCGAAC
GATGATTATCTAGCTTGCTAGATATGATTAGTGGCGGACGGGTGAGTAACATTTAGGAA
TCTGCCTAGTAGTGGGGGATAGCTCGGGGAAACTCGAATTAATACCGCATACGACCTAC
GGGTGAAAGGGGGCGCAAGCTCTTGCTATTAGATGAGCCTAAATCAGATTAGCTAGTTG
GTGGGGTAAAGGCCTACCAAGGCGACGATCTGTAACTGGTCTGAGAGGATGATCAGTC
ACACCGGAACTGAGACACGGTCCGGACTCCTACGGGAGGCAGCAGTGGGGAATATTGG

ACAATGGGGGCAACCCTGATCCAGCCATGCCGCGTGTGTGAAGAAGGCCTTTTGGTTGT
AAAGCACTTTAAGCAGGGAGGAGAGGCTAATGGTTAATACCCATTAGATTAGACGTTAC
CTGCAGAATAAGCACCGGCTAACTCTGTGCCAGCAGCCGCGGTAATACAGAGGGTGCG
AGCGTTAATCGGAATTACTGGGCGTAAAGCGAGTGTAGGTGGCTCATTAAGTCACATGT
GAAATCCCCGGGCTTAACCTGGGAACTGCATGTGATACTGGTGGTGCTAGAATATGTGA
GAGGGAAGTAGAATTCCAGGTGTAGCGGTGAAATGCGTAGAGATCTGGAGGAATACCG
ATGGCGAAGGCAGCTTCCTGGCATAATATTGACACTGAGATTCGAAAGCGTGGGTAGCA
AACAGGATTAGATACCCTGGTAGTCCACGCCGTAAACGATGTCTACTAGCCGTTGGGGT
CCTTGAGACTTTAGTGGCGCAGTTAACGCGATAAGTAGACCGCCTGGGGAGTACGGCCG
CAAGGTTAAAACTCAAAT

>RG6_ 16S ribosomal RNA gene

AGCCGCAGGTTCCCCTACGGCTACCTTGTTACGACTTCACCCTAGTCGCTGACTATACCG
TGGTCAAGGGTTTGAAACCTTGCCTTAAGGTAGAACCAACTCCCATGGTGTGACGGGCG
GTGTGTACAAGACCCGGGAACGCATTCACCGTGGCACGCTGATCCACGATTACTAGTAA
TTCCAGCTTCATGCACTCGAGTTGCAGAGTGCAATCCGAACTGAGGTATCTTTTAAGGAT
TTGCTTGACCTCGCAGTCATGCTTCCTGTTGTAGATACCATTGTAGCACGTGTGTAGCCC
AACACGTAAGGGCCATGAGGACTTGACGTCATCCCCACCTTCCTCCAGCTTATCACTGG
CAGTTCTTTCAGAGTGCCCAACTTAGTGATGGCAACTAAAAGTGAGGGTTGCGCTCGTT
GCGGGACTTAACCCAACATCTCACGACACGAGCTGACGACAGCCATGCAGCACCTGTGT
TTCGTCCGGCCGAACCGAAAACTCTAATCTCTTAGAGTCGCGACGAACATGTCAAGTGT
TGGTAAGGTTCCGCGCGTATCTTCGAATTAAACCACATGCTCCACTACTTGTGCGGGTCC
CCGTCAATTCATTTGAGTTTTAACCTTGCGGTCGTACTCCCCAGGCGGTGTGTTTATCGC
TTTCGCTGCGACACTGAAAATAAATTTCCAACGTCTAACACACATCGTTTACGGCATGG
ACTACGAGGGTATCTAATCCTCTTCGCTACCCATGCTTTCGTTCCTCAGTGTCAGTAATG
ATCCAGAAAGCCACCTTCGCAATTGGTATTCTTTCTAATATCTACGAATTTCACCTCTAC
ACTAGAAATTATGCTTTCCTCTATCATACTCTAGCTGAAAAGTTTTAATGGCAGTTCCAG
AGTTAAGCTCTGGGATTTCACCACTAACTTTTTCAACCACCTACGAACTCTTTAAGCCCA
GTAATTCCGAACTACGCTAGGTCCCTTCGTATTACCGCGGCTGCTGGCACGAAGTTAGC
CGGACCTT

>RG8_ 16S ribosomal RNA gene

GTAAACCTCTTTTAGCAGGGAAGAAGCGAAAGTGACGGTACCTGCAGAAAAAGCACCG
GCTAACTACGTGCCAGCAGCCGCGGTAATACGTAGGGTGCAAGCGTTGTCCGGAATTAT
TGGGCGTAAAGAGCTCGTAGGCGGTTTGTCGCGTCTGCTGTGAAAATCCGAGGCTCAAC
CTCGGACCTGCAGTGGGTACGGGCAAACTAGAGTGCGGTAGGGGAGATTGGAATTCCT
GGTGTAGCGGTGGAATGCGCAGATATCAGGAGGAACACCAATGGCGAAGGCAGATCTC
TGGGCCGTAACTGACGCTGAGGAGCGAAAGCGTGGGGAGCGAACAGGATTAGATACCC
TGGTAGTCCACGCCGTAAACGGTGGGAACTAGATGTGGGGTCCGTTCCACGGATTCCGT
GTCGCAGCTAACGCATTAAGTTCCCCGCCTGGGGAGTACGGCCGCAAGGCTAAAACTCA
AAGGAATTGACGGGGGCCCGCACAAGCGGCGGAGCATGCGGATTAATTCGATGCAACG
CGAAGAACCTTACCAAGGCTTGACATATAGAGGAAAAGTGTAGAAATACACTCCCCGC
AAGGTCTCTATACAGGTGGTGCATGGTTGTCGTCAGCTCGTGTCGTGAGATGTTGGGTTA
AGTCCCGCAACGAGCGCAACCCTCGTCCTATGTTGCCAGCACGTAATGGTGGGAACTCA
TGGGATACTGCCGGGGTCAACTCGGAGGAAGGTGGGGATGACGTCAAATCATCATGCC
CCTTATGTCTTGGGCTTCACGCATGCTACAATGGCCGGTACAGAGGGCAGCAATACCGC
AAGGTGGAGCGAATCCCAAAAAGCCGGTCTCAGTTCGGATTGAGGTCTGCAACTCGACC
TCATGAAGTTGGAGTCGCTAGTAATCGTGGATCAGCAACGCCACGGTGAATACGTTCCC
GGGCCTTGTACACACCGCCCGTCAAGTCATGAAAGTCGGTAACACCCGAAGCCGGTGGC
CCAACCGCAAGGAGGGAGCTGTCGAAGGTGGGATCGGTGATTAGGACTAAGTCGTAAC
AAGGTAGCCGTACCGGAAGGTGCGGCTGGATCACCT

Supplementary file 3. List of carbon metabolism-related genes and their annotations in the single-cell *Pelagibacter* spp. genomes from RG1 and RG6.

File S3 was showed in a separate Excel file.

Supplementary file 4. Identified beta-carotene 15,15'-dioxygenase genes from the single-cell *Pelagibacter* spp. genomes from RG1 and RG6.

>RG1_DGDANKKC_19687_ Beta-carotene 15,15'-dioxygenase

TTGGTTTGTTATACGTTGCTACAAAACCAATGGATAGAAAATAATATTTCCCTTTTATTT
TTTTGTTTTATTTTAATTTTAACTCTTGGTGTATCTCATGGCGCCTTAGATCATCTTCGAG
GTGAAAAAATTTTAAAGCCTATTTTAAAGAGCAGGTGGTTTTTGGTTTTTTATCCTGGCT
ATATTAGCCTTTCATTATTTGTCATTATTTGCTGGATATTATTTCCATCAATCTCATTATT
GATATTTCTTTTATTTGCAGGATACCATTTTGGCGAAGAGGATTTGAGTTTTTTTAAAGA
AGAGCAAGGTTTAATATTTAACATGACAGGGTTTTTAAAGGGATTTTTAATTATAACGTT
ATCGCTTCATTTTAATTTTGAGACCACTTCAACTTTTTTTAATTATCTGATGGTTGATATA
ACCCCTTATTCAGATCTGAAGTCTTATACCTCTATATTATTTGCTATAAATTTATTGTTAT
TGATTAGCGGTTTGATCTATTTATTTAAAAATCATTTAAATGAATTGGTTTTAATTTTACT
TGAAGTTATTCTTATTATTATCAGTTTTAAGTATTTGCCCTTGATTTTAGCTTTCACGCTT
TATTTTTGTTTTCTTCATTCATCAAAACATATCACTGGCCTTGCTAAAGAATTGGATGAT
GATGATTTGATTAATGGTTTTAGGTTATTTGCAAAAAAAGCAATTCCTTTAACCGTTTTA
ACAGGAATTGGTGCGTTAAGTGTTGTTTTTTTTCTCAATCATTCACTGACAGAAAACATT
ATCCAAACCATCTTTATAGGTTTAGCTTCACTTACTCTTCCCCATATATTATTAGAAGTG
ATAGATAAAAAATAA

>RG1_DGDANKKC_21827_ Beta-carotene 15,15'-dioxygenase

ATGCAAATTAAAAAAATACATTCTTATTTTTTAATAATATTTTCATTATTAGCTTTCATAT
TTTATTTATTAATCCGAAATAATCTAATTCAAGATAATCTTTATTTACTATTTTTTTCCTT
TTTTTTAATTCTAACCTTAGGTGTTTCGCACGGAGCCTTGGACCATCTGCGTGGCAAGAA
AATATTCTACCCTTTATTAAAACAAAGATGGTTTTTATTCTTTTATCCAGGTTATATCTTA
TTAAGTTTAATAGTTATTAGCAGCTGGGTTACATTTCCCACTATAACGTTATTATTATTTT
TGCTTATCGCCAGTTATCATTTTGGTGAAGAAGACCTTAGTTTTTTTTTAGAAAATAAGG
GAATATTTTTTAGTTTTATTAGCTTTCTAAAAGGATTGTTAATTATCACTGCATCATTTCA
TTTTAATTTTGAGACAACTGCATTATTTTTTCAATATTTATTTGTGCCAAGTGAAAATTAT
CAAACTTTAATTCACTATAAGACTTTGCTATTTTCGGTTAATTTAATTCTACTTGTAATTG

GCCTGATTAATTTACTTAAAAATCAAATTGATAAGCTAGTATTAATTTTGATGGAAGTCT
TATTGATTGTAATCTCTTTTAAATACTTACCTTTAATTTTAGCATTTACATTATACTTTTG
TTTTCTCCATTCATCTAAACATATCCTGGGGCTGTCTAAAGAATTAGATCCTGAAAATAT
TACTAATGGACTAAAACTCTTTGTCATTAAAGCTGCGCCACTGACCGCATTAACTGCTAT
TGCAGCTGTTTTATTTGTTATTTTATGGAGCGAAAGTATTAGTGAAAATATTATTAAAAC
AATCTTTATAGGTTTGGCTTCACTTACTCTTCCTCATATACTATTAGAAGTATTGGATAA
AAAATAA

>RG1_DGDANKKC_23847_Beta-carotene 15,15'-dioxygenase

TTGCTGATATTATTAATTGGCGTTACACATGGATCTCTAGATCATCTAAAAGGTAAAAA
ATTATTAAGTCATTATAATATAAGTAATACTTTTTATTTTTATATTGCTTATATCTTTATT
GCTTCTTTGATCATAATTTTATGGCTTATTTTTCCAGCTCAATTGTTAATTATTTTTCTAAT
TGTAGCTGCTTATCATTTTGGCAAGGAAGATACCCAATTTTTAGTACCTTACACTAGTTA
TACAAATCAATTATTATTTATACTAAAGGGTTCCCTGGTTATTTTTGCACCTTTATATTTT
AATTTTGCTGAAACAATAGAGTTATTTAAATTATTATTAATAGAGAATGAAAATTTTTAT
ACTCTGTTGGGTAAATTAGAAGAATTAAAAATATTTTTAATTGTTATTAGTTTAAGCACA
TTAGCAAGTCTATATCTGTTTTTAAAAAATTACGAAATCAATAAAGTAACAATTTTTATC
GACTATATATCAATTATTTTGATAAATTTTTACTTTTCTCCCCTAATAGCTTTTACAATAT
ACTTCTGTTTTTTACATTCTATTAGACACATTGTGAATTTAATTTTTGAATTAGATGAAGA
TGATTTTCTAAATGGAGCTAAATTATTTATAATTAAATCTGTTCCTCTAACAGCGCTTAC
AGGTTTATTAGCATTGCTTTCACTTTATTTTATCAATTTTAAAGTCATATTTGATGATGCA
ATTATTAAAGTCATTTTTATTGGTTTGGCGTCTTTAACCTTTCCTCATATATTGCTGGAAT
ATTTATTAGAAAAAAATGAAAAACAAAAAACTTAA

>RG6_HJAEHAFH_03893_Beta-carotene 15,15'-dioxygenase

ATGATAAAAAAAATTAATCTAACTCATTCCTTTATATTTTTTATAATTGTTAATTTATTTA
TTTTTTTAAATCTAATATTTTCTAATTTTTCTATTACACCATTTGGATGTTTGTTGCTGATA
TTATTAATTGGCGTTACACATGGATCTCTAGATCATCTAAAAGGTAAAAAATTATTAAGT
CATTATAATATAAGTAATACTTTTTATTTTTATATTGCTTATATCTTTATTGCTTCTTTGAT
CATAATTTTATGGCTTATTTTTCCAGCTCAATTGTTAATTATTTTTCTAATTGTAGCTGCT

TATCATTTTGGTAAGGAAGACACTCAATTTTTAATACCTTACACCAGTTATACAAATCAA
TTATTATTTATACTAAAAGGTTCTTTAATTATTTTTGCACCTTTGTATTTTAATTTTGCTG
AAACAATAGAGTTATTTAAATTATTATTAATAGAGAATGAAAATTTTTATACTCTGCTGG
GTAAATTAGAAGAATTAAAGATATTTTTAATAGGTGTTAGTTTAGGCTCATTAGCATGCT
TGTATCTATTTTTAAAAAATTATGAAATCAATAAAGTAACAATTTTTATTGATTACACTT
CAATTCTTTTGGTTAATTTTTATTTTTCACCTCTTGTGGCTTTTACAGTGTATTTTTGTTTT
TTACATTCTATTAGACACATTGTGAATTTAATTTTTGAATTAGATAAAGATAATTTCTTA
AATGGAGCCAAATTATTTATAATTAAATCTATTCCTCTAACAGTGCTTACAGGTTTATTA
GCATTGCTTTCACTTTATTTTATCAATTTTAAAGTCATATTTGATGATGCAATTATTAAAG
TCATCTTTATTGGTTTGGCGTCTTTAACTTTTCCTCATATATTGCTGGAATATTTATTAGA
AAAAAATGAAAAACAAAAAACTTAA

Supplementary file 5. Identified proteorhodopsin (PR) genes from the single-cell *Pelagibacter* spp. genomes from RG1 and RG6.

> RG1_DGDANKKC_01494 Green-light absorbing proteorhodopsin

ATGTCGCGATCGCAGGTCGACGCCGAATACCGGCCCGCGTTGATGGTATCTGGACTGGT
GGTATCGATTGCGTGTTACCACTACTTCATGATTCGTCACAGCTGGAATGATGCCTACAC
GCTTGCCGAGGGCGGGGCTGGTTACATCGGCACCGGTGCCGCGTTCAACGACTTTTACC
GCTATGCTGACTGGATACTGACAGTCCCACTGCTGATGGTTGAACTTGTTGCTGTCCTCC
GACTGCCGGCGGCGAAGGCGACGAGTCTGCTCACCCGGCTGGTGATTGCTGCTGCAGCA
ATGATTGCTCTCGGCTATCCCGGTGAAGTGATCGCTGATCCGAGCCGCTGGACTGAGCG
GGTTATCTGGGGAGGTTTGTCCTCAATTCCGTTCTTTTACATTCTCTACGTGCTGTGGGGT
GAGCTGACCAAGTCGCTTGACAGTCAGCCGCTTGCAGCCCGGAAGCTGATTGAGATTTG
CCGGCTTGTCTTGTTGATCACCTGGGCGGTCTATCCGATTGCCTATGCCTTGGGTGGCAC
GCCCGAGGCGCTCACCGCCAAGGCAGGTGGCGCAGTTGGTGCTGGTGGTGTGGTCGGCC
TGCAAATCGGGTACGCCATCGCCGATATGACCGCCAAGGCTGGCTTTGGCGTGTTGATC
TACTTCATTGCCCGTGCCAAGAGCAGTGGGTCGGGTGAGGCTGCTGTGGCTGCCGCCTG
A

> RG1_DGDANKKC_11844 Green-light absorbing proteorhodopsin_PR3 (94% homology to Alpha proteobacterium HIMB59, unclassified *Pelagibacteraceae*)

ATGTTTAAAATAAAAACCCTATTACCGGCATCTTTATTGTTGGTATTACCTCAGTTCGCA
AACGCTGCTGCGAACCTTGAATCCAACGATTTCGTTGGTATTTCATTTTGGCTAATTTCT
ATGGCCCTAGTCGCTTCAACAGCATTCTTCTTCTTAGAAACTCAAAGAGTTAGTGCTAAG
TGGAAAACTTCACTAACAGTATCTGGTCTAGTTACTTTAGTGGCTGCTGTTCACTATTTC
TATATGCGTGATGTATGGATCGCTACTGGCGATACACCAACCGTTTATAGATATATCGA
CTGGCTAATCACTGTTCCATTATTGATGGTTGAATTCTACATCATCCTAAGAGCTGTCGG
AAACGCATCTGCTGGTATCTTCTGGAGACTTATGATTGGTACTCTAGTAATGCTAGTTGC
TGGTTATATGGGTGAAGCTGGATACATCAACCTATGGGCAGGTTTCATCGTTGGTATGG
CTGGTTGGGCATATATCTTATATGAGGTATTTGCTGGTGAAGCTGGAAAAATGGCTGCT
GATAAAGCGCCTGCTTCAGTTCAATCTGCTTTCTCAACAATGAGATGGATTGTAACAATT

GGTTGGGCTATTTATCCTTTAGGATATTTCTTTGGTTATTTAACAGGCGGAGCAAGTATG
GAAGCTCTAAACGTTATCTATAACCTTGCTGACGTTCTAAATAAGATTGCATTCGGTGTA
ATCATCTGGAATGTTGCAACAACAGAATCTAAAGCTTAA

> RG1_DGDANKKC_12002 Green-light absorbing proteorhodopsin_RG4 (DNA no significant homology, translated peptide 59% homology to rhodopsin)

ATGACACCTCTCATCGCTGCAGCGGTCGAAACCGCCCTTTCTCTTCAGTTCATGGGCAAC
AGTCCGCTTGAGTCGATCACACTCTACGCTTTTTTTGCGACGACCATTGCGATGGGTGCT
GGAGCCGCCTTCTTCACGCTGATGCTCTTCCGTGGTGACCTCGACACCGAGCAGTACAC
CGTGGTGGCCCTTTCGGCCCTCATCTGTGGAATTGCCTGCGTCAACTACCAGCAGATGAC
TGGGGTCTATCAGGCGACCGGCGGTGGCTTTCCCACCGGGCTACGGTACATCGATTGGC
TGCTGACCACGCCCCTGCTGCTTTTGACCTTTCCGCTTCTGCTCGGGCTCGGCTGGGATT
CAATCAAGGTCTTCCTGCAACTGGTACTTCTTGATCTGGCGATGATTCTGCTCGGCTTCG
TGGGTGAGATCTCTCCGGTTGGGAGCCAGAACTGGTGGCTCTTCTTCGCCCTGAGCTGTG
GCTGTGGGGGGATGATTCTGCTGACGCTGTTCTTCTCGCTGGAGGAGGCGATTCATGAC
GCCCCAGTGGAACTGGCCAAGTCGCTGGAGACTCTTCGACTCTTTATCCTGATCGGCTG
GGCGGTCTATCCGCTCGGTTTCCTCTTGGCCCTCTCCGGCGACGCCACCCCACGGGAACT
GATCTACAACGTGGCCGATGTCATCAACAAGGTCGGCTTCGGCCTCGTCGTCCACGCCG
GCGTCATCAACTCGGTCGGCGGTTCAGGCTGGGGCCGGCGATAG

> RG1_DGDANKKC_29961 Blue-light absorbing proteorhodopsin

ATGATTGGTACATTAGTAATGCTAGTAGGTGGATACTTAGGAGAAGCAGGATACATCAA
CACTACACTTGGTTTCATTATCGGTATGGCTGGTTGGGTATACATTCTTTATGAAGTATT
CTCTGGTGAAGCAGGTAAGAGAGCAGCGAAAAGTGGTAACAAAGCACTTGTAACTGCT
TTTGGTGCAATGAGAATGATCGTTACAGTAGGTTGGGCTATTTACCCGTTAGGTTACATT
TTTGGTTACATGACAGGTGGAGTAGACGCTAGCTCACTAAACGTGATTTACAACGCAGC
TGACTTCTTGAACAAGATCGCTTTCGGTCTGATCATTTGGGCAGCAGCAATGCAACAAC
CTGGTAGAGCTAAGTAA

> RG6_HJAEHAFH_00563 Blue-light absorbing proteorhodopsin_RG2 (98% homology to

Candidatus *Pelagibacter* sp. HIMB1321, GenBank: LT840186.1)
ATGAAAAAACTTAAATTGTTTGCTCTTACAGCTGTTGCTCTTATGGGTGTTACAGGTGTA
GCAAACGCAGAAACTGCCATGTTAGCTCAAGATGATTTCGTAGGAATTTCATTTTGGCT
AGTATCAATGGCTTGTTTAGCTGCTACTGTGTTCTTCTTTCTAGAAAGAAGTTCGGTTCC
AGCTGGATGGAGAGTTTCAATGACAGTTGCTGGTCTAGTAACTGGTATTGCATTCGTAC
ACTACATGTACATGAGAGATGTATGGATCGCTACTGGTGACTCACCAACTGTTTATAGA
TACATTGACTGGTTAATTACTGTACCGTTATTGATGTTAGAATTCTATTTTGTTCTATCAG
CAGTAAACAAAGCAGACTCTGGAATTTTCTGGAGACTGATGATTGGTACATTGGTAATG
CTAGTAGGTGGATACTTAGGAGAAGCAGGATACATCAACACTACACTTGGTTTCATTAT
CGGTATGGCTGGTTGGGTATACATTCTTTATGAAGTATTCTCTGGTGAAGCAGGTAAGA
GAGCAGCGAAAAGTGGTAACAAAGCACTTGTAACTGCTTTTGGTGCAATGAGAATGATC
GTTACAGTAGGTTGGGCTATTTACCCGTTAGGTTACATTTTTGGTTACATGACAGGTGGA
GTAGACGCTAGCTCACTAAACGTGATTTACAACGCAGCTGACTTCTTGAACAAGATCGC
TTTCGGTCTGATCATTTGGGCAGCAGCAATGCAACAACCTGGTAGAGCTAAGTAA

> RG6_HJAEHAFH_00938 Green-light absorbing proteorhodopsin_RG1 (DNA no significant homology, translated peptide is similar to PR)
ATGATTGAAGGCGTCTTGTTGTTGGGCGGAGTAATGCCCGACTTGTCGGTTTTTCAGTAC
AACGCAGTTGACAACATTTTCTCGATGACGGTCGCGACGATGGGGGCGGCGGCCCTCTT
TCTGTTCATGTCCCGTTCGCAGGTTGATGCGGAATATCGGCCGGCGCTGATGGTGTCGG
GATTGGTTGTTTCGATCGCCTGCTATCACTATTTCATGATCCGGCACAGCTGGAATGATG
CCTACACGTTGGCCGAGTCGGGAAGTCAGTATGTTGGTACTGGAGCCGCTTTCAACGAC
TTCTATCGATACGCAGACTGGATTTTGACAGTGCCCCTGCTGATGGTCGAACTCGTTGCC
GTGCTGCGATTGCCTGCCGCGAAGGCGACGAGCCTGCTCACGCGGCTGGTCGTTGCCGC
CGCTGCGATGATCGCGCTGGGCTACCCCGGAGAAGTTATTGCCGACCCGAATCGGTGGA
CCGAACGAGTTGTCTGGGGCGGGCTTTCTTCGGTGCCCTTCTTCTACATCCTCTACGTGC
TTTGGGTCGAATTGACCAAGTCGCTTGACAGCCAGCCTCCCGCGGCCCGGAGGCTCATC
GAGGTCTGCCGCCTCGTATTGCTGATCACTTGGGCGGTTTATCCGATCGCCTATGCGCTC
GGTGGGACCCCCGACGCGCTCGCGGCGAAAGCGGGAGGGCAGGTTGGTGCCGGTGGGA
TTGTCGGATTGCAGATTGGCTACGCCATTGCCGACATGACGGCAAAGGCGGGCTTCGGC

GTGCTGATCTACTTCATCGCCCGGGCGAAGAGCGGGACAGGCTCCGCAGACAGTGCGGC
TGTTGCAACTGCCTGA
