# Supplementary material for: Revealing CO2-Fixing SAR11 Bacteria in the Ocean by Raman-Based Single-Cell Metabolic Profiling and Genomics
Source: Biodes Res. 2022 Oct 13;2022:9782712. doi: 10.34133/2022/9782712 (PMC10521720; doi:10.34133/2022/9782712)
Supplement: Supplementary Materials — Supplementary file 1: identified 16S rRNA genes from single-cell Pelagibacter spp. genomes from RG1 and RG6. Supplementary file 2: identified 16S rRNA genes obtained from MDA products of SAGs from the RAGE-Seq samples. Supplementary file 3: list of carbon metabolism-related genes and their annotations in the single-cell Pelagibacter spp. genomes from RG1 and RG6. Supplementary file 4: identified beta-carotene 15,15′-dioxygenase genes from the single-cell Pelagibacter spp. genomes from RG1 and RG6. Supplementary file 5: identified proteorhodopsin (PR) genes from the single-cell Pelagibacter spp. genomes from RG1 and RG6. [file 9782712.f1.zip › Supplementary Information.pdf]

Supplementary information

Revealing CO_2-fixing SAR11 bacteria in the ocean by Raman-based single-cell metabolic profiling and genomics

Xiaoyan Jing[1,5,6,†], Yanhai Gong[1,6,†], Teng Xu[1, 6,†], Paul A. Davison[4], Craig MacGregor-Chatwin[4], C. Neil Hunter[4], La Xu[3], Yu Meng[1,6], Yuetong Ji[1,6], Bo Ma[1,6], Jian Xu[1,5,6,*], Wei E. Huang[2,*]

[1]Single-Cell Center, CAS Key Laboratory of Biofuels, Shandong Key Laboratory of Energy Genetics and Shandong Institute of Energy Research, Qingdao Institute of BioEnergy and Bioprocess Technology, Chinese Academy of Sciences, Qingdao, Shandong, China.

[2]Department of Engineering Science, University of Oxford, Parks Road, OX1 3PJ, Oxford, United Kingdom.

[3]Disease and Fishery Drugs Research Center, Marine Biology Institute of Shandong Province, Qingdao, Shandong, China.

[4]Department of Molecular Biology & Biotechnology, University of Sheffield, Western Bank, Sheffield S10 2TN.

[5]Laboratory for Marine Biology and Biotechnology, Qingdao National Laboratory for Marine Science and Technology, Qingdao, Shandong, China

[6]University of Chinese Academy of Sciences, Beijing, China.

[†]These authors contributed equally to this work.

*Corresponding author:

Wei E. Huang and Jian Xu

Email: wei.huang@eng.ox.ac.uk and xujian@qibebt.ac.cn

Materials and methods

Measurement of seawater environmental parameters

Seawater and relevant environmental parameters (**Table S1**), including water temperature (T), salinity, pH, dissolved oxygen (DO) and chemical oxygen demand (COD) were recorded *in situ* employing portable devices (YSI Pro_Plus, Yellow Springs, OH, USA). Other environmental parameters, such as total phosphorus (TP), total nitrogen (TN), orthophosphate (PO_4^{3-}-P) and chlorophyll *a* (Chl-*a*) were assayed in laboratory according to Standard Methods (1). All data were collected in accordance with the approved Chinese regulations and standards.

Bacterial species, media and growth conditions for the cross-feeding verification experiment

The series of mock microbiota include two bacteria of *Synechococcus elongatus* PCC7942 and *Micrococcus luteus* OY14 (isolated from marine environment in our laboratory), and one fungus of *Saccharomyces cerevisiae* BY4742. *S. elongatus* PCC7942 was cultured in the BG11 medium under light at 28°C, diluted to OD 600 of ~0.5 and then inoculated at a ratio of 1:50 into 4 mL BG11. *M. luteus* OY14 was cultured in Luria-Bertani (LB) medium (Tryptone, Yeast extract, NaCl, pH 7.0) at 37°C, diluted to OD 600 of ~0.5 and then inoculated at a ratio of 1:50 into 4 mL of LB medium. *S. cerevisiae* BY4742 was cultured in YPD medium (Yeast Extract, Peptone, glucose, pH 6.5~6.8) at 30°C, diluted to OD 600 of ~0.5 and then inoculated at a ratio of 1:50 into 4 mL of YPD. To prepare the media for ^{13}C labeling, each of the microorganisms was incubated in its medium respectively until reaching the logarithmic phase, washed using distilled water, and then mixed to form the synthetic consortia in an 1:1:1 ratio. The synthetic consortia were incubated under $NaH^{13}CO_3$ (98 atom% 13 C, Sigma-Aldrich, Canada; 2 mM final concentration in BG11) at room temperature for 24 h, and then subject to single-cell Raman spectroscopy, respectively.

Multiple displacement amplification

The REPLI-g Single Cell Kit (Qiagen, Germantown, MD, USA) was used for the single cell amplification according to the manufacture's instruction. Cell lysis was carried out at 65°C for 15 min with 2 μl lysis buffer for each sample, followed by addition of 1 μl stop solution to neutralize the lysis buffer. REPLI-g sc Reaction Buffer and REPLI-g sc DNA Polymerase were added and the mixture was incubated at 30°C for 8 hours with 70°C hot-lid temperature for MDA reactions. Blank control

(without any cells) was also included to detect and quantify potential contamination. After that, the MDA products were processed for 16S rRNA gene PCR analysis using 27F and 1492R primers (**Table S3**). Once the MDA products of single cell genomic DNA have been confirmed to contain single species 16S-rRNA, they were sent out for further high throughput sequencing.

Library construction and next generation sequencing

For 16S rRNA sequencing: Total genome DNA from the filter membranes was extracted using Magen Hipure Soil DNA Kit (Lot: HE280200) according to the manufacturer's protocols. DNA was quantified using the Qubit® 3.0 Fluorometer (Invitrogen, Carlsbad, CA, USA). For each sample, 20-30 ng DNA was used to generate amplicons using a MetaVx™ Library Preparation kit (Genewiz, South Plainfield, NJ, USA). V3 and V4 hypervariable regions of prokaryotic 16S rDNA were selected for generating amplicons and subsequent taxonomy analysis. The V3 and V4 regions were amplified using forward primers containing the sequence "CCTACGGRRBGCASCAGKVRVGAAT" and reverse primers containing the sequence "GGACTACNVGGGTWTCTAATCC". At the same time, indexed adapters were added to the ends of the 16S rDNA amplicons to generate indexed libraries ready for downstream NGS sequencing on Illumina Miseq. PCR reactions were performed in triplicate 25 μL mixture containing 2.5 μL of TransStart Buffer, 2 μL of dNTPs, 1 μL of each primer, and 20 ng of template DNA. DNA libraries concentration were validated by Qubit® 3.0 Fluorometer. Sequencing was performed on an Illumina MiSeq instrument in paired-end layout (PE250) according to manufacturer's instructions (Illumina, San Diego, CA, USA) by GENEWIZ.

For single-cell genome sequencing: The target MDA products were treated with S1 Nuclease (Thermo Fisher Scientific, Waltham, MA, USA) to degrade the single-stranded nucleic acids, and then purified by Agencourt AMPure XP Beads (Beckman Coulter, Brea, CA, USA). Next generation sequencing library preparations were constructed following the manufacturer's protocol (NEBNext® Ultra™ DNA Library Prep Kit for Illumina®). For each sample, 1 μg genomic DNA was randomly fragmented to <500 bp by sonication (Covaris S220). The fragments were treated with End Prep Enzyme Mix for end repairing, 5' Phosphorylation and dA-tailing in one reaction, followed by a T-A ligation to add adaptors to both ends. Size selection of Adaptor-ligated DNA was then performed using AxyPrep Mag

PCR Clean-up (Axygen), and fragments of ~410 bp (with the approximate insert size of 350 bp) were recovered. Each sample was then amplified by PCR for 8 cycles using P5 and P7 primers, with both primers carrying sequences which can anneal with flowcell to perform bridge PCR and P7 primer carrying a six-base index allowing for multiplexing. The PCR products were cleaned up using AxyPrep Mag PCR Clean-up (Axygen), validated using an Agilent 2100 Bioanalyzer (Agilent Technologies, PaloAlto, CA, USA), and quantified by Qubit2.0 Fluorometer (Invitrogen, Carlsbad, CA, USA).

Then libraries with different indexes were multiplexed and loaded on an Illumina HiSeq instrument according to manufacturer's instructions (Illumina, San Diego, CA, USA). Sequencing was carried out using a 2x150 paired-end (PE) configuration; image analysis and base calling were conducted by the HiSeq Control Software (HCS) + OLB + GAPipeline-1.6 (Illumina) on the HiSeq instrument. Samples were quantified using a Qubit® 2.0 Fluorometer (Invitrogen, Carlsbad, CA, USA).

Data analysis

For 16S rRNA sequencing: The software package of QIIME 2 (2) was used for 16S rRNA data analysis. The forward and reverse reads were joined using VSEARCH with minimum overlap of 20 bp. Quality filtering on joined sequences was performed with default criteria. Then the sequences were further quality controlled using a 16S positive filter of Deblur workflow, and discarded features appearing less than twice across all samples. Alpha and beta diversity were also analyzed using QIIME 2 ("diversity core-metrics-phylogenetic"). Taxonomic classification was performed using q2-feature-classifier plugin with pre-fitted sklearn-based taxonomy classifier (classify-sklearn; pretrained on GreenGenes database with 99% OTUs). Relative abundances of the bacterial taxa at the phylum, class, order, family, genus and species levels were calculated and compared, respectively.

For single-cell genome sequencing: A dedicated pipeline was built (https://github.com/gongyh/nf-core-scgs) to efficiently analyze SAG datasets by integrating various tools with Nextflow (3). Briefly, reads that passed illumina's chastity filter were first quality checked using FastQC, and then quality trimmed using Trim Galore in paired end mode for each sample. To detect contaminated DNA fragments, clean reads were phylogenetically classified using Kraken (4). Clean reads were then assembled into contigs using SPAdes (5) in single-cell mode, taxonomic composition of assembled contigs (longer than 200 bp) were visualized using BlobTools (https://f1000research.com/articles/6-

1287/v1), annotation of assembled genomes were conducted using Prokka (6), KofamKOALA (7) and eggNOG-mapper (8). Considering the inevitable DNA contaminations for environmental samples, assembled contigs were further split into bins by taxonomic annotations (in family level) for each SAG, followed by completeness estimation using CheckM (9). Bins with completeness larger than 50% or the highest in corresponding sample were chosen for subsequent analysis and visualized using a t-SNE plot (based on 4-mer frequency profilings). For selected bins, KEGG Orthology (KO) for each gene was extracted using custom scripts and further used to mining potential carbon fixation associated pathways.

Benchmarking of RAGE-Seq

To gauge the possibility of contamination, we have benchmarked RAGE-Seq based on one-cell-resolution sorting of the carotenoid-Raman-peak phenotype from a mock microbiota (10). Specifically, we have constructed a mock microbial community that consists of *Synechococcus elongatus* PCC7942 (Se), *Escherichia coli* K-12 DH5α (Ec), *Helicobacter pylori* ATCC26695 (Hp), and *Saccharomyces cerevisiae* BY4742 (Sc) mixed in an 1:1:1:1 ratio. Experiments in triplicate (R1, R2 and R3) were designed to test the specificity of sorting and sequencing carotenoid-producing cells (note that in this mock community, only Se contains carotenoids). In total, 10 successful one-cell RAGE-Seq reactions of this mock microbiota were performed. Our results suggest that, without any exception, the top contig bin from each of such precisely one-cell assemblies corresponds to the target cell, i.e., the single-cell 16S rRNA sequencing based genotype is always consistent with the SCRS-predicted phenotype (e.g., whether the cell harbors pigments). Therefore, the possibility of contamination in the RAGE-Seq process appears to be low. This experiment from mock microbiome supports the assignment of the top contig bin of one-cell RAGE-Seq to the target cell for the actual samples (**Fig. S9**).

References

1. APHA. 1998. Standard Method for the Examination of Water and Wastewater. 20th Edition, American Public Health Association, Washington DC,.
2. Caporaso JG, Kuczynski J, Stombaugh J, Bittinger K, Bushman FD, Costello EK, Fierer N, Peña AG, Goodrich JK, Gordon JI, Huttley GA, Kelley ST, Knights D, Koenig JE, Ley RE, Lozupone CA, McDonald D, Muegge BD, Pirrung M, Reeder J, Sevinsky JR, Turnbaugh PJ, Walters WA, Widmann J, Yatsunenko T, Zaneveld J, Knight R. 2010. QIIME allows analysis of high-throughput community sequencing data. Nature Methods 7:335-336.
3. Di TP, Chatzou M, Floden EW, Barja PP, Palumbo E, Notredame C. 2017. Nextflow enables reproducible computational workflows. Nature Biotechnology 35:316.
4. Wood DE, Salzberg SL. 2014. Kraken: ultrafast metagenomic sequence classification using exact alignments. Genome Biology 15:R46.
5. Anton B, Sergey N, Dmitry A, Gurevich AA, Mikhail D, Kulikov AS, Lesin VM, Nikolenko SI, Son P, Prjibelski AD. 2012. SPAdes: a new genome assembly algorithm and its applications to single-cell sequencing. Journal of Computational Biology 19:455-477.
6. Torsten S. 2014. Prokka: rapid prokaryotic genome annotation. Bioinformatics 30:2068-9.
7. Aramaki T, Blanc-Mathieu R, Endo H, Ohkubo K, Kanehisa M, Goto S, Ogata H. 2019. KofamKOALA: KEGG ortholog assignment based on profile HMM and adaptive score threshold. Bioinformatics 36:2251-2252.
8. Huerta-Cepas J, Forslund K, Coelho LP, Szklarczyk D, Jensen LJ, Von MC, Bork P. 2016. Fast Genome-Wide Functional Annotation through Orthology Assignment by eggNOG-Mapper. Molecular Biology & Evolution 34:2115.
9. Parks DH, Imelfort M, Skennerton CT, Hugenholtz P, Tyson GW. 2015. CheckM: assessing the quality of microbial genomes recovered from isolates, single cells, and metagenomes. Genome Research 25:1043-1055.
10. Jing X, Gong Y, Xu T, Meng Y, Han X, Su X, Wang J, Ji Y, Li Y, Jia Z, Ma B, Xu J. 2021. One-Cell Metabolic Phenotyping and Sequencing of Soil Microbiome by Raman-Activated Gravity-Driven Encapsulation (RAGE). mSystems doi:10.1128/mSystems.00181-21:e0018121.
