# Supplementary material for: Revealing CO2-Fixing SAR11 Bacteria in the Ocean by Raman-Based Single-Cell Metabolic Profiling and Genomics
Source: Biodes Res. 2022 Oct 13;2022:9782712. doi: 10.34133/2022/9782712 (PMC10521720; doi:10.34133/2022/9782712)
Supplement: Supplementary Materials — Supplementary file 1: identified 16S rRNA genes from single-cell Pelagibacter spp. genomes from RG1 and RG6. Supplementary file 2: identified 16S rRNA genes obtained from MDA products of SAGs from the RAGE-Seq samples. Supplementary file 3: list of carbon metabolism-related genes and their annotations in the single-cell Pelagibacter spp. genomes from RG1 and RG6. Supplementary file 4: identified beta-carotene 15,15′-dioxygenase genes from the single-cell Pelagibacter spp. genomes from RG1 and RG6. Supplementary file 5: identified proteorhodopsin (PR) genes from the single-cell Pelagibacter spp. genomes from RG1 and RG6. [file 9782712.f1.zip › Table SI.pdf]

Supplementary Table S1. Properties of the seawater sampled from Yellow Sea of China.

	**Sample: #Primary***
**Sample area**	Laoshan Bay
**Sample type**	Seawater from euphotic zone
**Location**	Latitude (N), 36° 15′; Longitude (E), 120° 41′
**Collection time**	May 11, 2019, 16:30 pm
**Depth (m)**	10.800
**Temperature (℃)**	16.900
**Salinity (PSS-78)**	30.475
**Dissolved oxygen (mg/L)**	8.320
**Chemical oxygen demand (mg/L)**	1.700
**Total nitrogen (mg/L)**	1.266
**Ammonia nitrogen (mg/L)**	0.020
**NO_3^--N (mg/L)**	0.024
**NO_4^--N (mg/L)**	0.800
**Total phosphorus (mg/L)**	0.400
**Orthophosphate (g/L)**	4.630
**Chlorophyll** *a* (g/L)	1.560
**pH**	8.670

*primary sample control.

Supplementary Table S2. Numbers of 16S rRNA sequencing reads for microbial diversity analysis of each sample.

Sample	Raw reads	Processed reads	Pielou's evenness	Observed OTUs	Phylogenetic Diversity	Shanno
**12C-CO_2-01**	105862	51159	0.919	753	27.068	8.787
**12C-CO_2-02**	127586	61739	0.915	738	27.964	8.721
**12C-CO_2-03**	116038	55463	0.926	719	27.189	8.791
**12C-HCO_3-01**	107000	50326	0.909	668	29.731	8.525
**12C-HCO_3-02**	122924	58915	0.933	779	31.341	8.962
**12C-HCO_3-03**	125404	60220	0.919	735	28.322	8.748
**13C-HCO_3-01**	115396	54962	0.920	686	27.162	8.667
**13C-HCO_3-02**	135634	64887	0.922	759	28.289	8.818
**13C-HCO_3-03**	115178	55450	0.916	734	28.588	8.719
**C-combind-01**	140134	67946	0.913	746	27.851	8.715
**C-combind-02**	139208	67326	0.912	725	25.725	8.661
**C-combind-03**	120988	58297	0.933	744	28.434	8.896
**C_free_01**	178864	85784	0.907	720	25.933	8.613
**C_free_02**	151218	72488	0.925	765	30.940	8.864
**C_free_03**	138474	66230	0.917	741	30.744	8.743

Supplementary Table S3. Primers used in 16S rRNA gene PCR amplification.

Description	Name	Primer Sequence (5'→ 3')
**16S rRNA, full length**	27F	AGAGTTTGATCCTGGCTCAG
	1492R	GGTTACCTTGTTACGACTT

Supplementary Table S4. Sequencing and assembly statistics for the functional single-cell genomes.

**Sample**	**Sequencing**		**Assembly**		
	Raw reads	Clean reads	Assembly size	Number of contigs	N50
RG1	17.91 M	17.90 M	36.37 Mb	38,704	2.47 kb
RG5	16.66 M	16.65 M	2.36 Mb	1,388	11.35 kb
RG6	16.89 M	16.88 M	6.79 Mb	6,429	3.04 kb
RG8	18.16 M	18.15 M	1.43 Mb	2,528	1.21 kb

Supplementary Table S5. Taxonomy assignment of the targeted cell based on NCBI/NT or GTDB. Bold font: taxonomic classification of the target cell. Non-bold font: taxonomic classification of the contaminant or the presumable symbiotic bacteria.

RAGE-Seq-derived one-cell SAG	Class (Genus) assignment based on BLASTN of NCBI/NT	GTDB-tk taxonomy for each bin
**RG1**	*Pelagibacteraceae*	d__Bacteria;p__Proteobacteria;c__Alphaproteobacteria;o__HIMB59;f__HIMB59;g__;s__
	Planctomycetaceae	d__Bacteria;p__Planctomycetota;c__Planctomycetes;o__Pirellulales;f__UBA1268;g__;s__
	Alteromonadaceae	d__Bacteria;p__Proteobacteria;c__Gammaproteobacteria;o__Enterobacterales;f__Alteromonadaceae;g__Alteromonas_A;s__
**RG5**	*Moraxellaceae*	d__Bacteria;p__Proteobacteria;c__Gammaproteobacteria;o__Pseudomonadales;f__Moraxellaceae;g__Moraxella_A;s__Moraxella_A osloensis_A
**RG6**	*Pelagibacteraceae*	d__Bacteria;p__Proteobacteria;c__Alphaproteobacteria;o__Pelagibacterales;f__Pelagibacteraceae;g__Pelagibacter;s__
	Planctomycetaceae	d__Bacteria;p__Planctomycetota;c__Planctomycetes;o__Pirellulales;f__UBA1268;g__;s__
**RG8**	*Microbacteriaceae*	d__Bacteria;p__Actinobacteriota;c__Actinomycetia;o__Actinomycetales;f__Microbacteriaceae;g__Pontimonas;s__
