# Supplementary figures and images for: Revealing CO2-Fixing SAR11 Bacteria in the Ocean by Raman-Based Single-Cell Metabolic Profiling and Genomics
Source: Biodes Res. 2022 Oct 13;2022:9782712. doi: 10.34133/2022/9782712 (PMC10521720; doi:10.34133/2022/9782712)

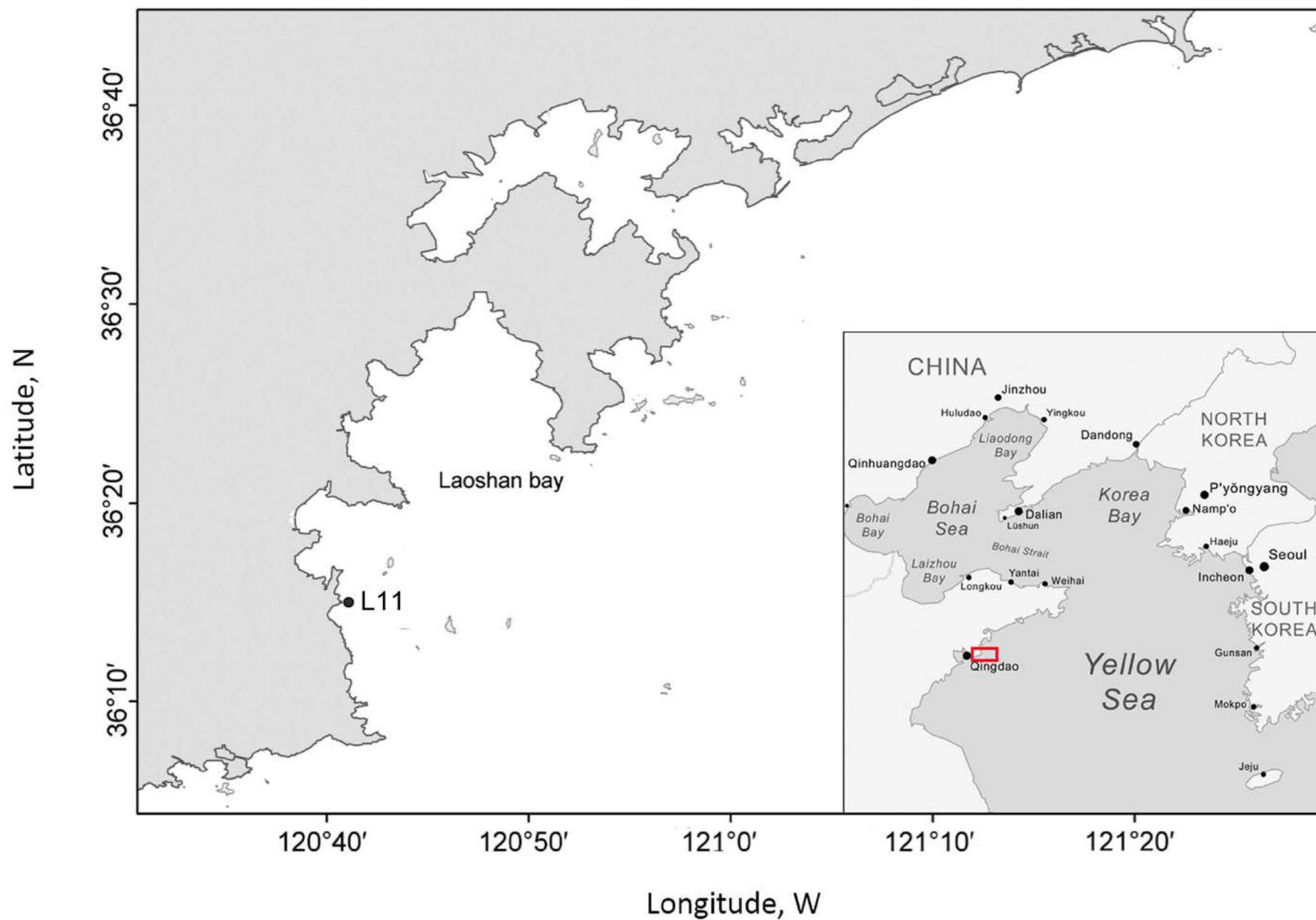

**Figure S1**

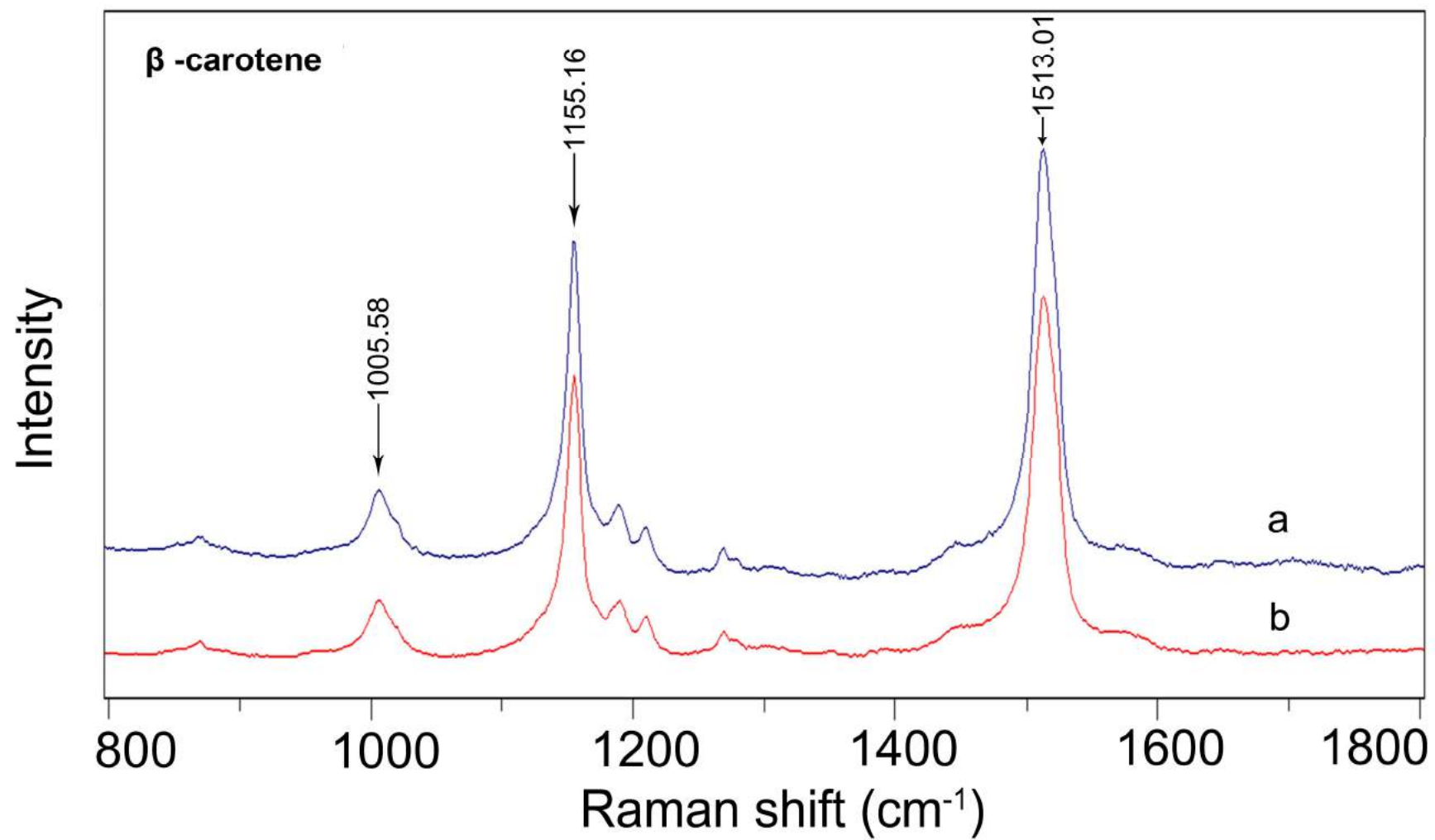

**Figure S2**

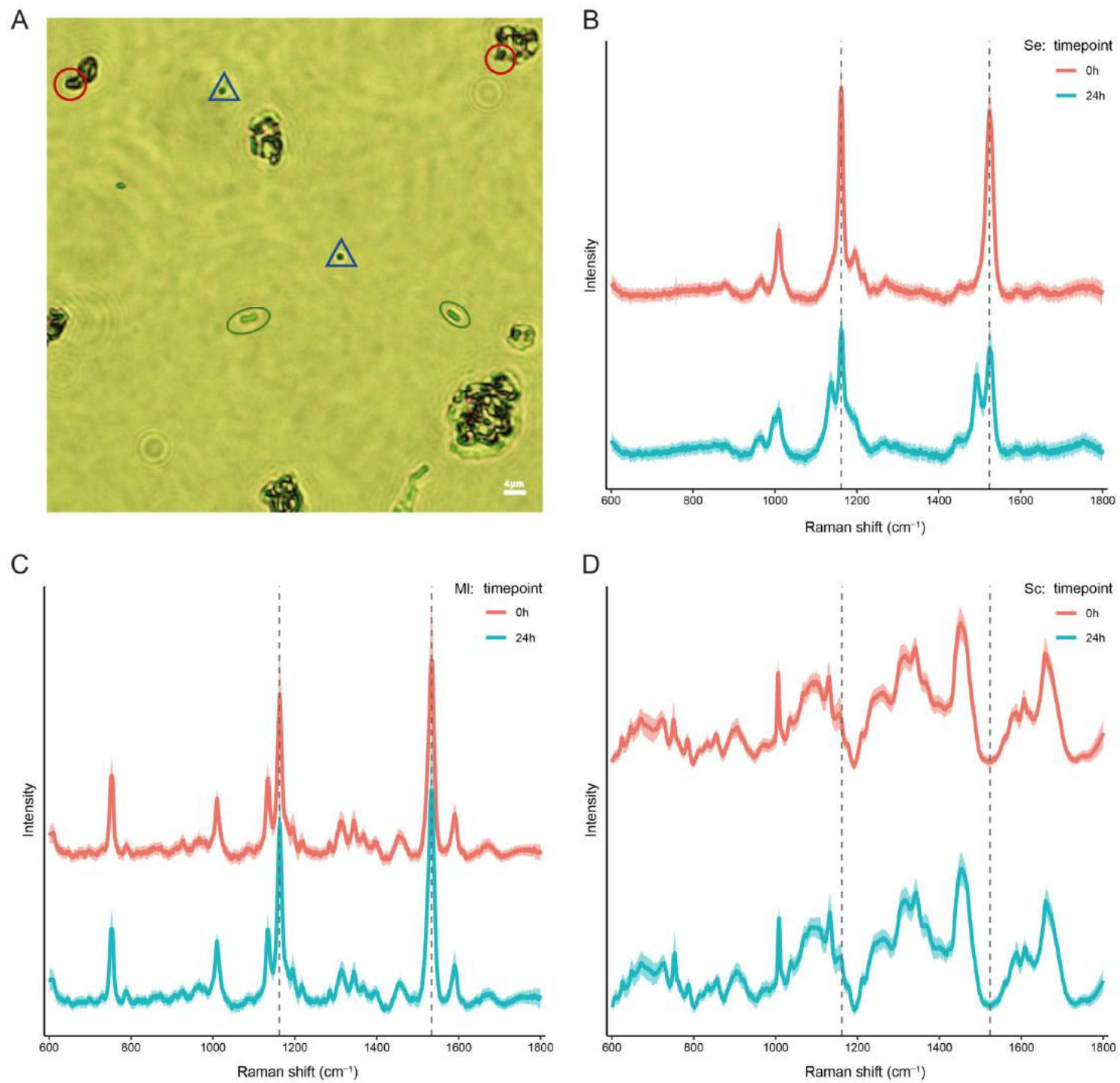

**Figure S3**

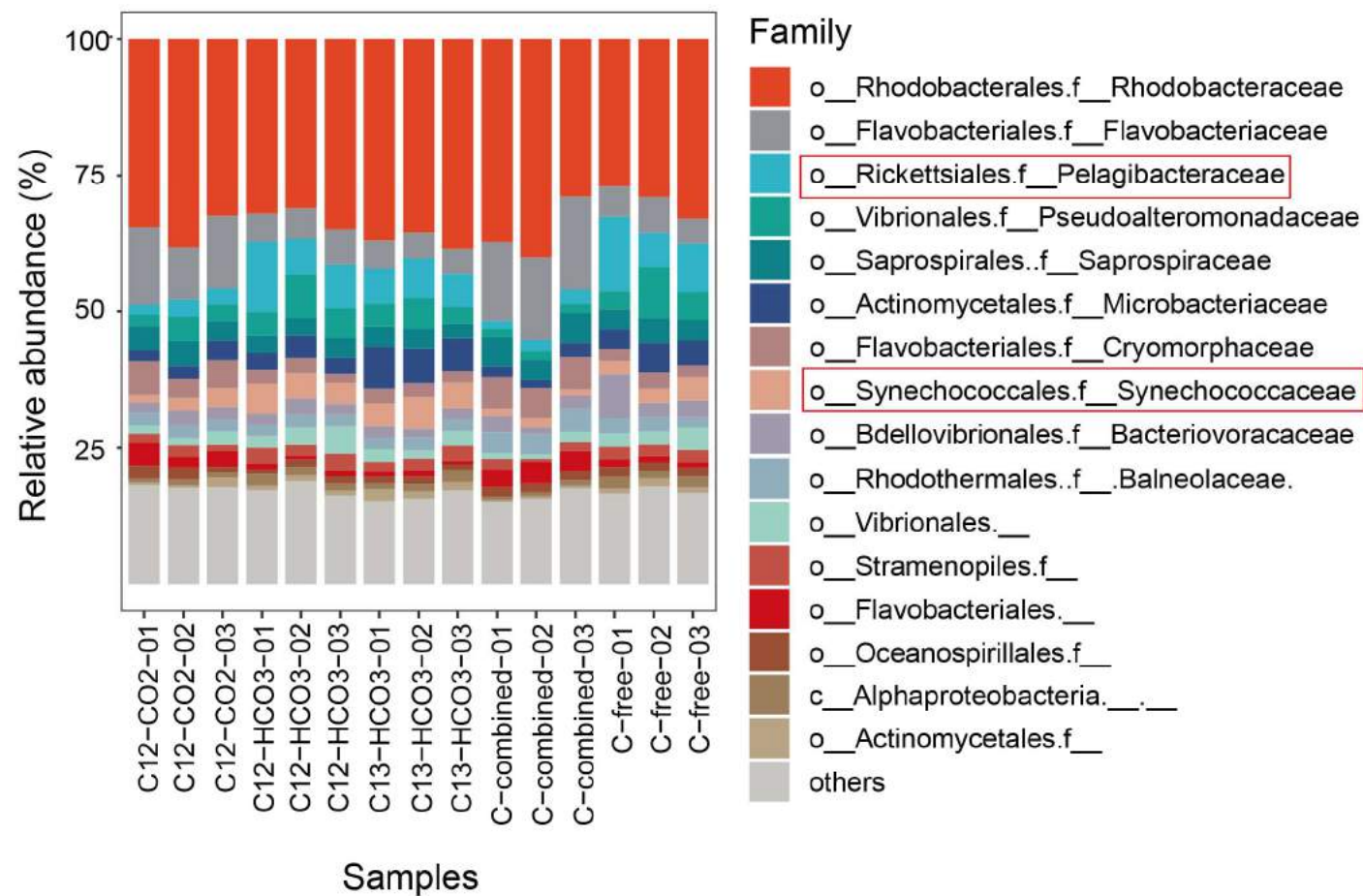

Figure S4A

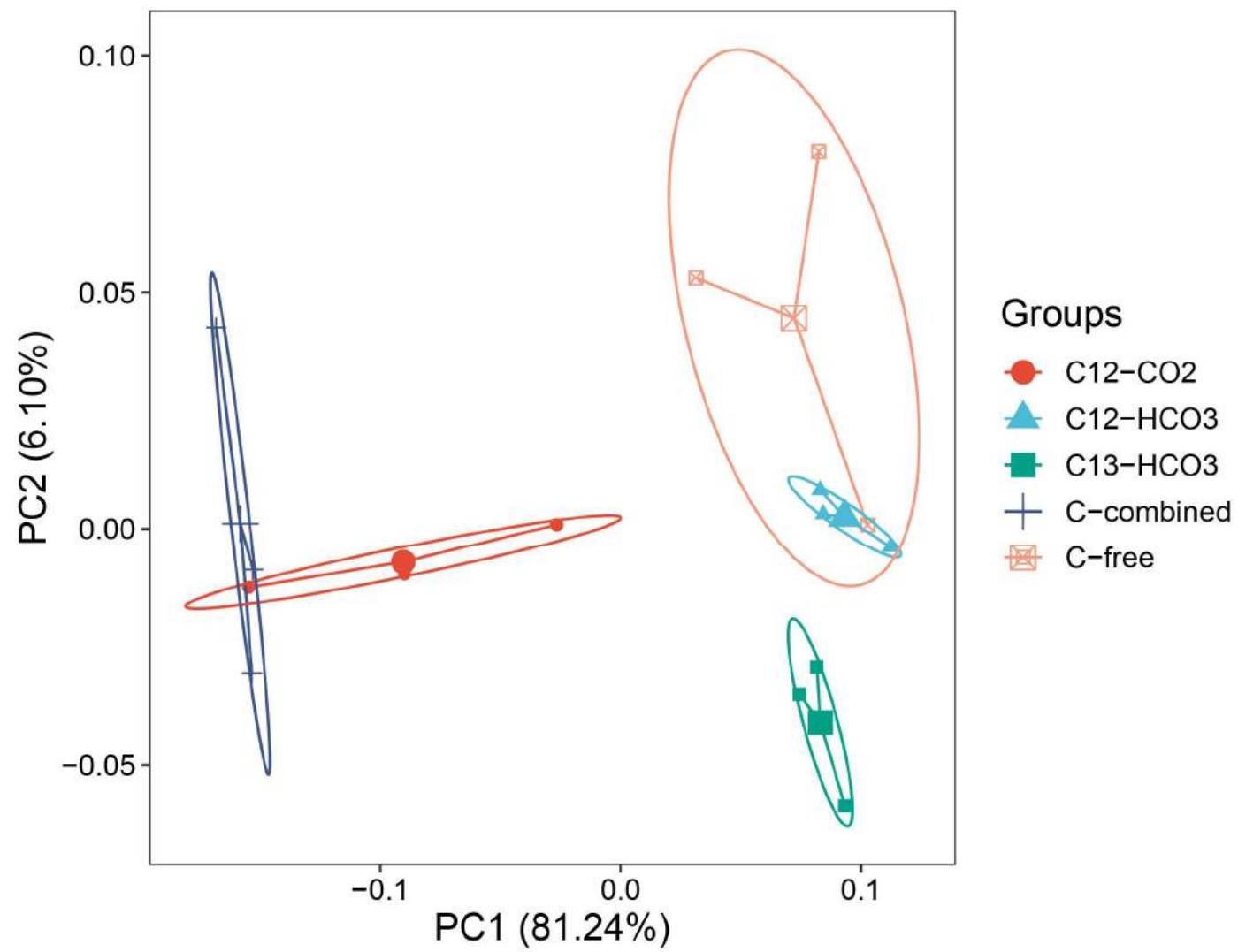

**Figure S4B**

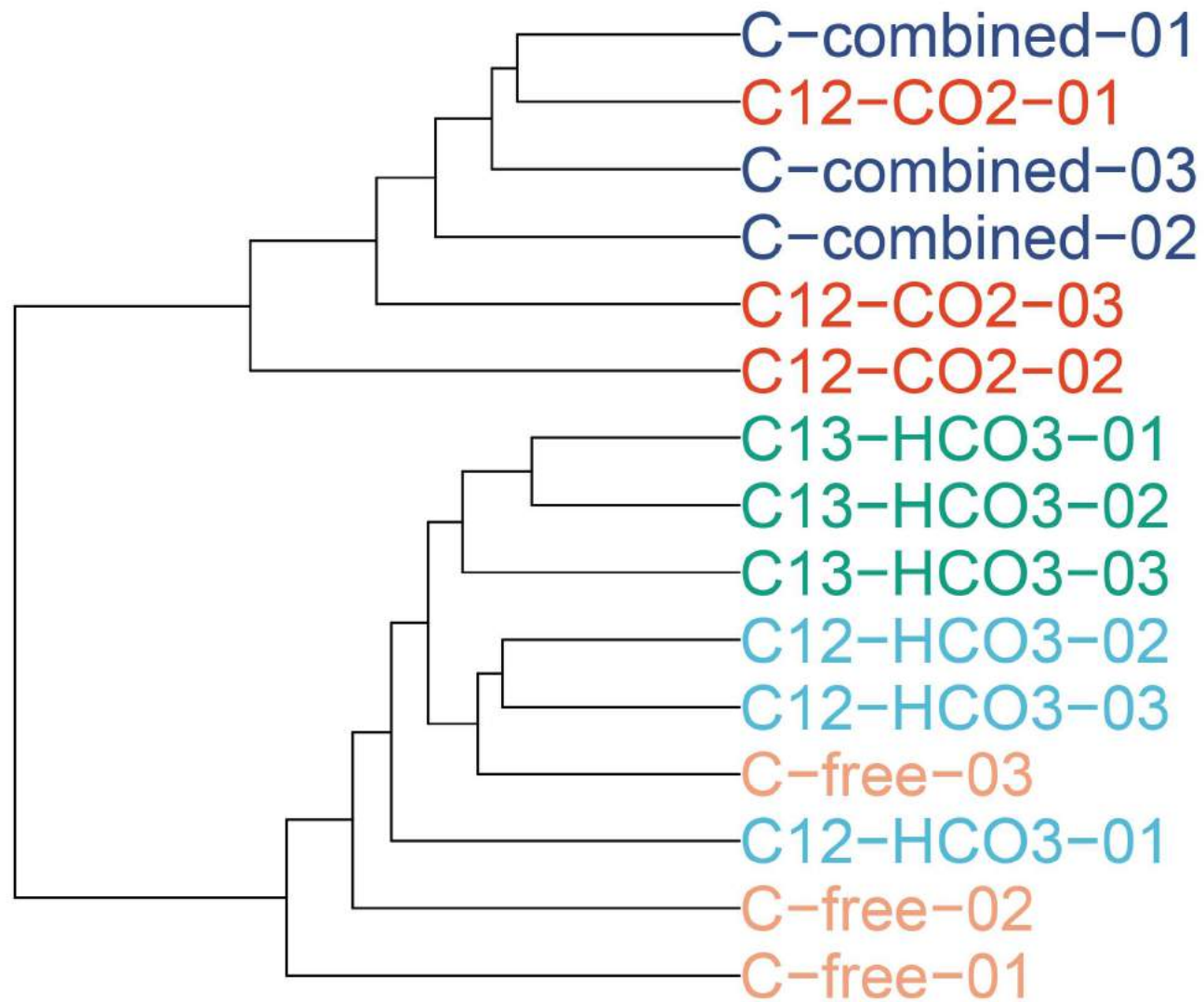

**Figure S4C**

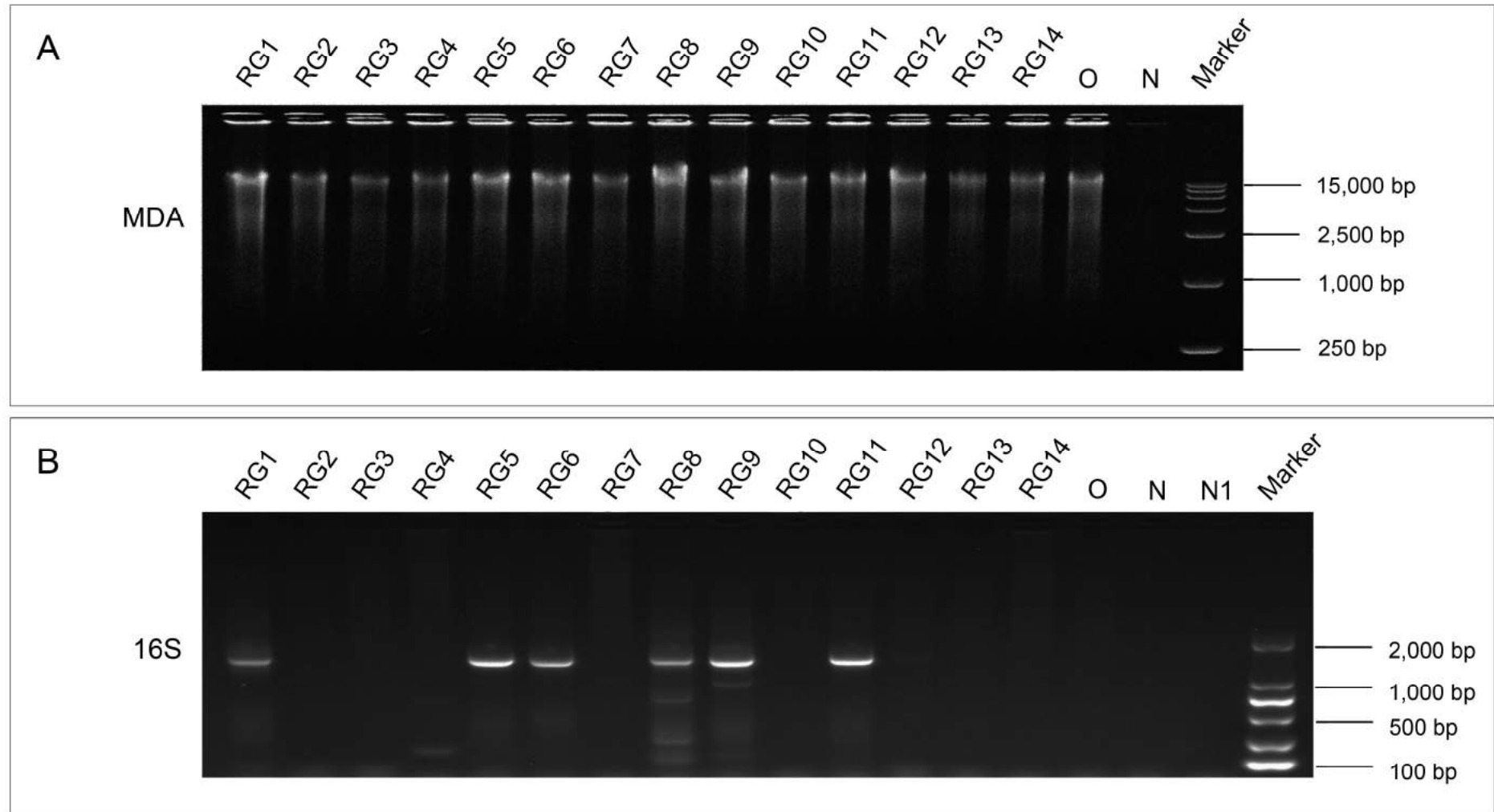

**Figure S5**

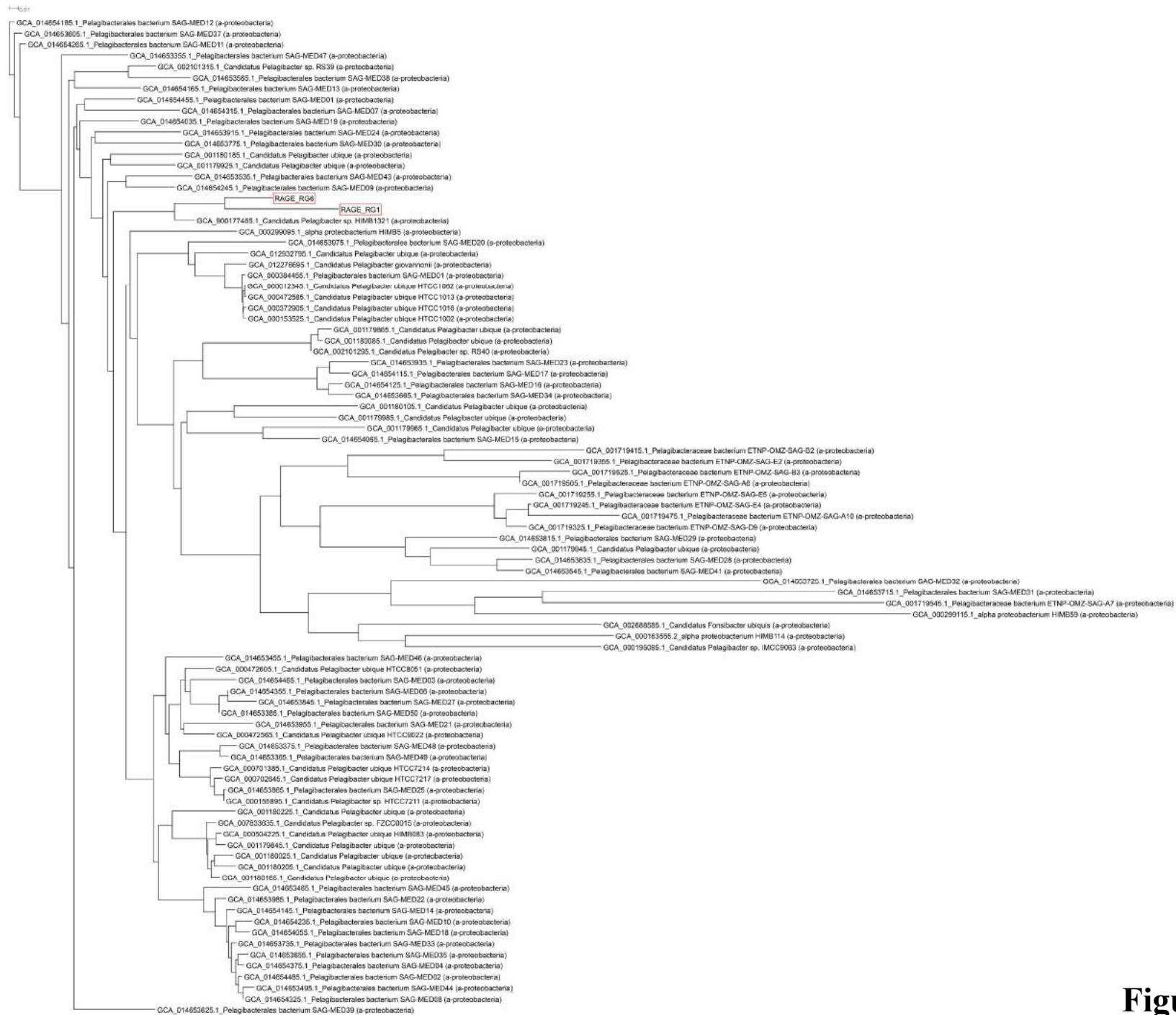

**Figure S6**



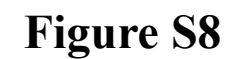

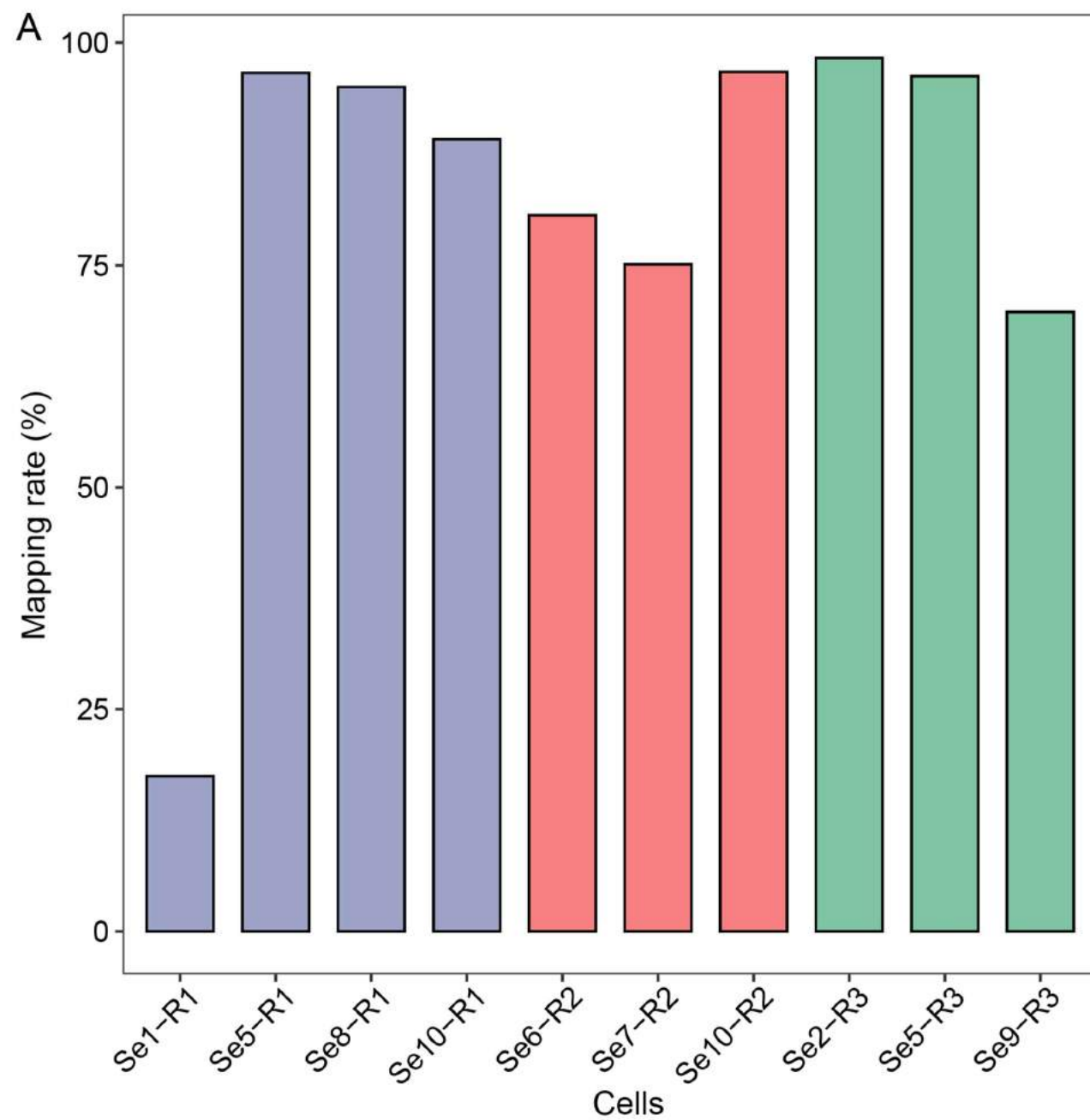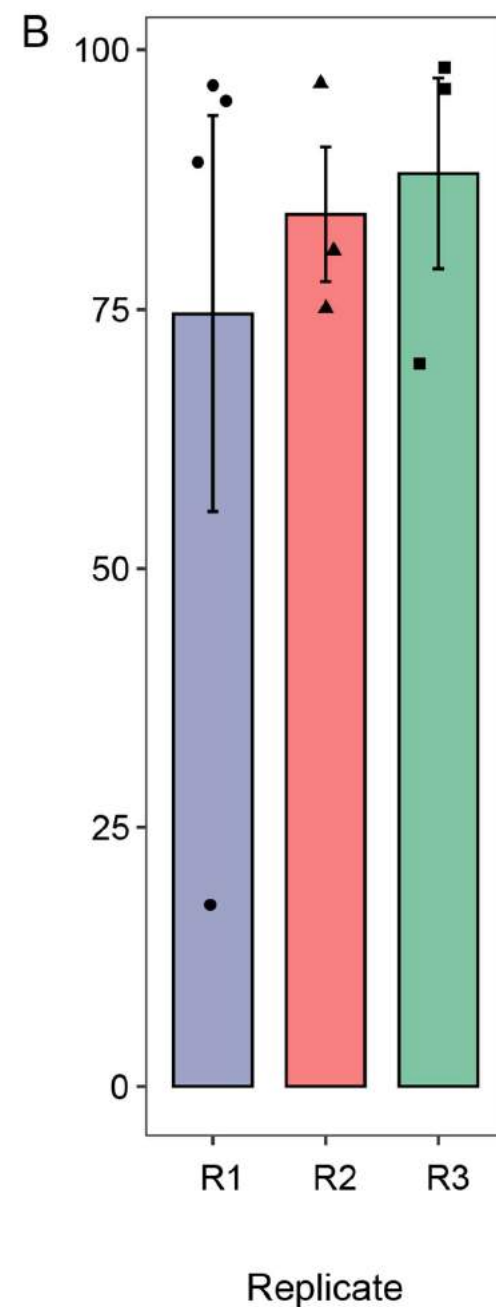

**Figure S9**

Supplement: Supplementary Materials — Supplementary file 1: identified 16S rRNA genes from single-cell Pelagibacter spp. genomes from RG1 and RG6. Supplementary file 2: identified 16S rRNA genes obtained from MDA products of SAGs from the RAGE-Seq samples. Supplementary file 3: list of carbon metabolism-related genes and their annotations in the single-cell Pelagibacter spp. genomes from RG1 and RG6. Supplementary file 4: identified beta-carotene 15,15′-dioxygenase genes from the single-cell Pelagibacter spp. genomes from RG1 and RG6. Supplementary file 5: identified proteorhodopsin (PR) genes from the single-cell Pelagibacter spp. genomes from RG1 and RG6. [file 9782712.f1.zip › Figure SI.pdf]
